# Poor-tasting pediatric medicines: Part 1. A scoping review of their impact on patient acceptability, medication adherence, and treatment outcomes

**DOI:** 10.3389/fddev.2025.1553286

**Published:** 2025-04-22

**Authors:** Sejal R. Ranmal, Jennifer Walsh, Catherine Tuleu

**Affiliations:** ^1^ UCL School of Pharmacy, University College London, London, United Kingdom; ^2^ Sciense Ltd., London, United Kingdom; ^3^ Jenny Walsh Consulting Ltd., Nottingham, United Kingdom

**Keywords:** children, medicines, bitter, taste, palatability, acceptability, adherence, outcomes

## Abstract

**Background:**

Many medicines for children taste bitter and unpleasant, presenting a significant barrier to effective pharmacotherapy. Anecdotally, this issue is widely recognized; however, empirical evidence on the consequences of unpalatable medicines remains scarce and fragmented. The objective of this scoping review was to investigate the impact of poor tasting pediatric medicines on patient acceptability, medication adherence, and/or treatment outcomes.

**Methods:**

A literature search was performed in MEDLINE/PubMed, EMBASE and CINAHL from inception to June 2023. Eligibility criteria included interventional or observational studies conducted in children aged 0–18 years (population), administered an unpalatable oral medicine (exposure), with any reported impact on patient acceptability, medication adherence, and treatment effects (outcomes). Study screening and data extraction was completed using a standardized form on Covidence.

**Results:**

After searching 2,282 citations and reviewing 429 full-text papers, 225 articles were included in the final analysis. The impact of poor-tasting medicines was observed across 77 diseases or indications, with 156 different unpalatable medicinal products identified. Outcomes were most frequently linked to reduced patient acceptability, with 64% of articles reporting rejection responses, the need for strategies to aid administration (from positive reinforcement to physical restraint and forced administration), and impacts on prescribing practices (e.g., use of non-first line alternative therapies). Medication adherence impacts were reported in 27% of the reviewed studies, where poor taste was reported as a barrier to adherence in chronic diseases and correlated with incomplete dose administration in acute conditions. A small number of studies linked palatability with treatment outcomes, including viral suppression in HIV and seizure control in epilepsy.

**Conclusion:**

This review highlights the widespread adverse impact of poor-tasting pediatric medicines on patient experiences and outcomes, though the true extent of the issue may still be underreported. The problem affects children worldwide, across all age groups, and is frequently noted by parents, caregivers, and healthcare professionals in both clinical and domiciliary settings. These findings emphasize the need for the development and prescription of more palatable medicines for children, as well as the advancement of more universal taste-masking strategies to address this widespread problem.

## 1 Introduction

Globally, approximately 30% of the population, or around 2.4 billion people, are babies, children, and adolescents under the age of 18 years (United Nations, 2022). Almost all of this pediatric population will need to take medicines at some point during this stage of life, be it over-the-counter (OTC) products for minor, acute ailments to regular prescribed medicines to manage chronic diseases. Two important concepts must be considered when developing and prescribing medicines: patient acceptability, namely, *the overall ability and willingness of the patient to use and its care giver to administer the medicine as intended* (European Medicines Agency, 2013)*;* and medication adherence, *the degree to which use of medication by the patient corresponds with the prescribed regimen* (World Health Organization, 2003). Even the most efficacious drug treatments will be futile if patients do not take them as required.

Palatability is a crucial factor that impacts patients’ experiences of taking medicines. Regulatory agencies define this as *the overall appreciation of an (often oral) medicinal product in relation to its smell, taste, aftertaste and texture (i.e., feeling in the mouth)* (European Medicines Agency, 2013) and the *quality of a drug product that makes it pleasant or acceptable* in terms of these attributes (Food and Drug Administration, 2018). However, developing palatable medicines is challenging due to a major obstacle: many drugs have bitter and unpleasant tastes (Mennella et al., 2013). This is unsurprising, as most drugs are xenobiotics - chemical substances not naturally produced or expected to be present within the body - that can exert biological, and potentially adverse effects (Schiffman et al., 2002). Thus, bitterness is an innately aversive sensation that serves as an evolutionary, biological warning to prevent the ingestion of potentially toxic and harmful substances (Breslin, 2013; Wooding et al., 2021). Despite this natural aversion, it is crucial that patients are able to take their medications for effective clinical treatment.

Indeed, patients of all ages are more likely to prefer better-tasting medicines with favorable sensory and organoleptic properties, but this issue is particularly challenging in children. While adults are often able to tolerate unpalatable medicines due to their understanding of the health benefits, children’s experiences are shaped by their cognitive development and more primal sense of what is acceptable or not. Young patients may lack the ability to fully understand the importance of taking medication or cooperate in its administration. Taste perception is a complex process, and sensitivity and responses to the same stimuli can vary considerably among individuals. This can be influenced by a multitude of confounding factors including age, sex, genetics, ethnicity, and health status (Shizukuda et al., 2018; Cattaneo et al., 2022). Taste perception first develops *in-utero* and matures with age, though children have a recognized preference for sweet tastes and dislike for bitter stimuli which stems from their innate biology (Mennella and Bobowski, 2015; Ustun et al., 2022; Mennella, 2014). Studies have shown that children are more sensitive to bitter tastes compared with adults when exposed to the same stimulus (Ranmal et al., 2023; Mennella et al., 2005; Mennella et al., 2010).

**FIGURE 1:**
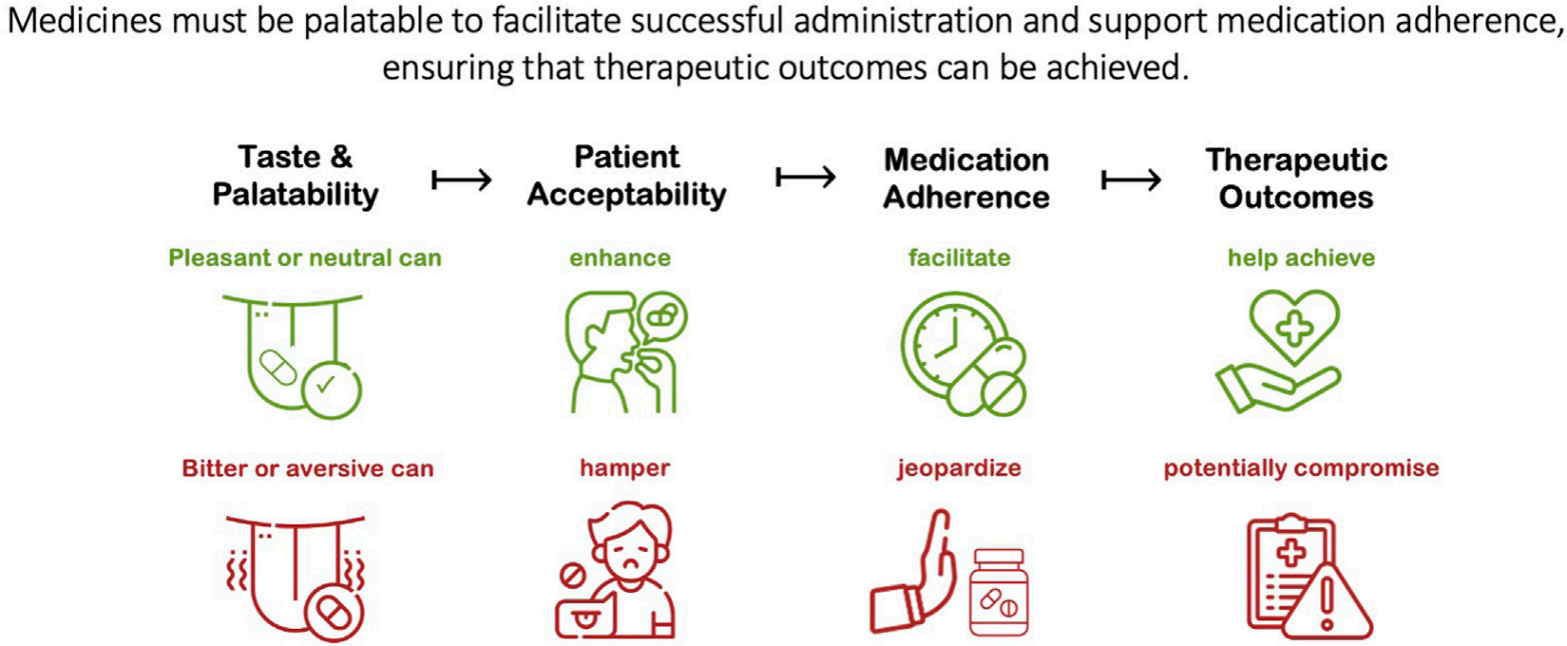
The relationship between medication palatability and patient acceptability, which in turn can impact medication adherence and the safety and efficacy of the treatment.

In its seminal guideline on the pharmaceutical development of pediatric medicines, the European Medicines Agency (EMA) acknowledges that *palatability is one of the main elements of the patient acceptability,* and in turn, *patient acceptability is likely to have a significant impact on patient adherence and consequently, on the safety and efficacy of a medicinal product* (European Medicines Agency, 2013). The United States Food and Drug Administration (FDA) also notes this association between palatability and patient acceptability, and the resulting impact on adherence, *a key factor in successful therapeutic intervention* (Food and Drug Administration, 2018). This proposed relationship is illustrated in Figure 1, showing in turn how medication taste can facilitate or compromise these related outcomes. This relationship is logically sound and anecdotally, the issue is widely recognized; however, empirical evidence demonstrating the impact of bad-tasting medicines is scarce and fragmented.

The objective of this scoping review was to investigate the impact of poor tasting pediatric medicines on three key concerns: patient acceptability, medication adherence, and treatment outcomes. A scoping review allowed this broad research question to be explored by systematically searching and synthesizing existing knowledge, while mapping key concepts and identifying gaps in evidence (Colquhoun et al., 2014). Insights into the real-world experiences of pediatric patients and their caregivers also provides valuable understanding of the practical challenges faced in administering unpalatable medicines. This is complemented by an accompanying article on the experiences of pediatric healthcare providers and caregivers in the United States and Sub-Saharan Africa (El-Sahn et al., 2025).

The conduct and reporting of the review was guided by the JBI Manual for Evidence Synthesis (Peters et al., 2020) and Preferred Reporting Items for Systematic Reviews and Meta-Analyses extension for scoping reviews (PRISMA-ScR) (Tricco et al., 2018). In line with the Population-Concept-Context (PCC) framework recommended by JBI, the review focused on: 1) population: children aged 0–18 years; including studies with patients, caregivers, and healthcare professionals (HCPs) involved in medicines administration; 2) concept: exposure to a bad tasting or unpalatable oral medicinal product impacting patient acceptability and administration, medication adherence, and/or therapeutic outcomes; and 3) context: any disease area or ailment and across all geographical locations.

## 2 Methods

The scoping review protocol was developed *a priori* and the PRISMA-ScR checklist is included in Supplementary Table S1.

### 2.1 Eligibility criteria

The review considered primary research from interventional studies (e.g., non-randomized or randomized controlled trials and uncontrolled studies) and observational studies (e.g., cross-sectional, cohort, and case-control). All study designs were eligible, including those using qualitative methodologies (e.g., groups or interviews) or quantitative methods (e.g., surveys, semi-structured questionnaires etc.). Published journal articles, conference abstracts, and letters were included, while reviews, editorials, and commentaries were excluded. When the same data were reported in more than one publication (e.g., a conference abstract and subsequent journal article), only the source reporting the most complete data set was used. No limits on date, subject or type were placed on the database search; however, articles published in languages other than English were excluded.

### 2.2 Information sources and search strategy

An initial limited search of two electronic databases, MEDLINE and Embase, was undertaken to identify key articles on the topic and optimize the search strategy. The Population-Exposure-Outcome (PEO) framework was used to outline the keywords and index terms for the full search (Supplementary Table S2). The full search was implemented in three electronic databases from inception to 17 June 2023: MEDLINE via PubMed (biomedical sciences, 1946–present), Embase via Ovid (biomedical and pharmaceutical sciences, 1947-present), and Cumulative Index to Nursing and Allied Health Literature (CINAHL) via EBSCO (nursing and allied health; 1981–present). The search strategy was tailored to the specific requirements of each database and was not limited by study design or year (see Supplementary Table S3). The full electronic search strategy used in MEDLINE/PubMed is exemplified below:

((((((pediatric) OR (child)) OR (infant)) OR (adolescent)) AND (((((((palatab*) OR (taste)) OR (smell)) OR (texture)) OR (mouthfeel)) OR (bitter)) OR (avers*))) AND ((((drug therapy) OR (drug formulation)) OR (drug dosage form)) OR (medication))) AND ((((adherence) OR (medication compliance)) OR (accept*)) OR (treatment outcome)) Filters: English.

### 2.3 Study screening and selection

All citations were collated, screened, and reviewed within Covidence, a web-based collaboration software platform that streamlines the production of systematic and other literature reviews (Covidence, 2023). The relevance of studies as sources of evidence was assessed using a two-stage screening process: 1) title and abstract screening and 2) full text review. Selection was performed against pre-specified inclusion or exclusion criteria based on the PEO framework and study characteristics. The eligibility criteria were uploaded into the software for reference during the screening. During the title and abstract screening, studies were excluded if they were conducted in adult populations, or if the study was in relation to food, beverages, nutritional supplements, or placebo formulations. To meet eligibility criteria, abstracts had to explicitly mention palatability and include an outcome related to the study objectives. The eligibility screening process for full text screening is outlined in Section 2.5. Both stages were completed independently by two reviewers (SRR and JW). Reviewers met throughout the screening process to resolve conflicts and discuss any uncertainties related to study selection. Cohen’s Kappa coefficient (κ) was calculated within Covidence as a statistical measure used to quantify the level of agreement between two main reviewers. Any disagreements were solved by consensus or by the decision of a third reviewer (CT).

### 2.4 Data extraction and charting

Data from selected studies was extracted independently by the two main reviewers using a data extraction tool developed within Covidence (see Supplementary Table S4). The tool was initially piloted by each reviewer on a random sample of studies and was iteratively modified by consensus as the charting process evolved. Following full data extraction, one reviewer (SRR) verified all charting to ensure accuracy and consistency of data.

Key information collated included 1) study characteristics including location countries categorized by geography and income group based on World Bank Group classifications (World Bank, 2024) and overall classification in relation to palatability report and type of outcome(s); 2) population age range categorized into six pediatric age groups proposed in regulatory guidance (European Medicines Agency, 2006) 3) exposure to medicinal product with disease area categorized by WHO International Classification of Diseases (ICD-11) the international standard for recording and reporting diseases and health related conditions (World Health Organization, 2022) and chemical substance categorized by WHO Anatomical Therapeutic Chemical (ATC) classification (WHO Collaborating Centre for Drug Statistics Methodology, 2023) or as “other” for unclassified interventional products used for treatment or diagnosis.

For studies that included both pediatric and adult participants, the mean or median age of the cohort was considered, and studies were excluded if this exceeded 18 years. In cases where this age information was not reported, the study was only included if the age range of participants started from 12 years old. This cutoff was chosen to account for differences in population definitions, as studies that had been conducted in adults were captured during the screening process when the inclusion criterion was from the age of 16 years.

### 2.5 Appraisal and synthesis

The process of study appraisal and analysis in relation to palatability and outcome reports is outlined in Supplementary Table S5. Primarily, poor taste or palatability had to be reported either formally (e.g., rated on scale by study participants) or informally (e.g., as a problem reported by authors). Different terms were used to describe these evaluations across studies including “palatability,” “acceptability,” “degree of liking,” “tolerance,” and “satisfaction;” however, the descriptions in the manuscript had to demonstrate that this was in relation to the sensory attributes of the medicinal product. Some studies described bitter taste as an adverse event (AE), and in these cases, further clarity was sought from product literature to determine if this was a taste disturbance related to the systemic effect of the active pharmaceutical ingredient (API) or sensory properties of the drug itself. Since there are no established thresholds or criteria to define whether a medicine is deemed palatable or unpalatable, the review included studies reporting any negative rating (e.g., less than neutral response on self-report scale) or negative response from any single participant in the study.

Impacts on patient acceptability and administration were categorized as rejection responses (e.g., resistance or spitting out), strategies to aid administration (e.g., mixing with food or drink) and any impacts on prescribing (e.g., drug therapy changes). Adherence outcomes had to include formal measures (e.g., pill counts or self-report missed doses) or studies exploring barriers to adherence (e.g., using questionnaire tools or qualitative interviews), where palatability was specifically reported as an obstacle. Studies were excluded if the authors simply remarked that palatability can influence acceptability or adherence, but no quantifiable outcomes were reported. Impact on therapeutic outcomes were included if the authors explicitly linked findings with poor palatability of the medicine and its impact on acceptability and adherence. The synthesis included quantitative analysis (e.g., frequency and proportions) of the PEO criteria and qualitative review of themes and content. The methodological quality or risk of bias of the included articles was not appraised, in accordance with guidelines for conducting scoping reviews (Peters et al., 2020).

## 3 Results

### 3.1 Selection of sources of evidence

**FIGURE 2:**
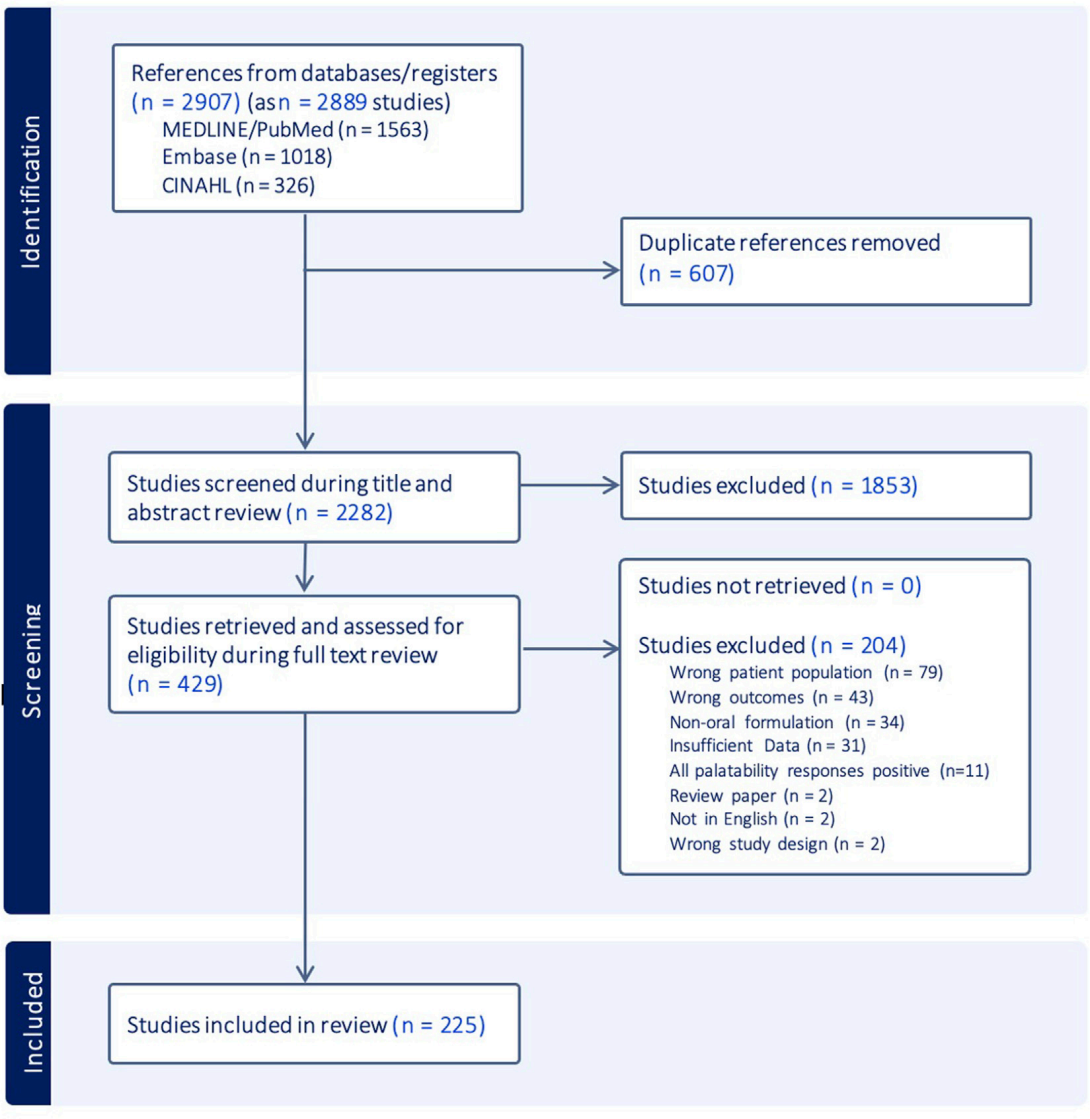
PRISMA flow diagram mapping information flow through the different phases of the scoping review.

The PRISMA flow diagram in Figure 2 illustrates the screening and selection process. A total of 2,907 citations (2,889 studies) were identified through the database search. After removing duplicates, 2,282 studies were screened based on titles and abstracts, followed by full-text review of 429 papers. Of these, 204 were excluded for not meeting eligibility criteria, with the most common reasons being incorrect study population, insufficient outcome reporting, or non-oral administration routes. Ultimately, 225 papers were included in the final scoping review analysis (Abdulla et al., 2010; Abu-Khalaf et al., 2018; Adams et al., 2013; Al-Ani et al., 2016; Aljebab et al., 2018; Allen et al., 2003; Almenrader et al., 2007; Ameen et al., 2006; Angelilli et al., 2000; Angwa et al., 2020; Ansah et al., 2001; Bagger-Sjoback and Bondesson, 1989; Baka-Ostrowska et al., 2021; Baker et al., 2021; Barnett and Bhatt, 2020; Bartoli et al., 2006; Berg et al., 2006; Bergene et al., 2019; Bertholet-Thomas et al., 2021; Beuter et al., 2019; Block et al., 2005; Block et al., 2006; Braga et al., 2015; Bryson, 2014; Buchanan et al., 2012; Bullington et al., 2007; Bunupuradah et al., 2006; Caglayan et al., 1989; Cañete et al., 2010; Celebi et al., 2009; Chadwick et al., 2005; Cheng, 2004; Christiansen et al., 2014; Chung et al., 2000; Cifaldi et al., 2004; Claes et al., 2014; Cloyd et al., 1992; Coetzee et al., 2015; Coetzee et al., 2016; Cohen et al., 2005; Cohen et al., 2009; Cote et al., 2002; Cronin et al., 2016; Dagan et al., 1994; Dagnone et al., 2002; Dalton et al., 2019; Davies et al., 2008; Dawson and Sharpe, 1993; DelRosso et al., 2020), (Dillman et al., 2018; Di Pierro et al., 2014; Duniva Inusa et al., 2021; El Edelbi et al., 2015; Elgammal et al., 2022; Escobedo et al., 2008; Escoda et al., 2017; Ewing et al., 2015; Frange et al., 2018; Freedman et al., 2010; Gamston, 2013; Garcia-Prats et al., 2022; Gaskell, 2016; Gibson et al., 2010; Giralt et al., 2019; Goldberg et al., 2013; Goode et al., 2003; Gotesman et al., 2020; Goyanes et al., 2019; Greenley et al., 2018; Greenwood and Vallabhaneni, 2015; Gutierrez-Colina et al., 2018; Gyedu et al., 2023; Hames et al., 2008; Hansen et al., 2008; Haslund-Krog et al., 2022; Hirsch-Moverman et al., 2021; Hoffstedt et al., 2023; Hofmanová et al., 2020; Holas et al., 2005; Holloway et al., 2018; Holmberg, 1994; Horodniceanu et al., 2017; Hou et al., 2022; Ibrahim and Al Ansari, 2016; Imanieh et al., 2022; Isik et al., 2008; Jaffé and Grimshaw, 1983; Jahnsen and Thorn, 1987; Jain et al., 2013; Jamieson et al., 2021; Joanne and Ibrahim, 2018; Kedenge et al., 2013; Kekitiinwa et al., 2016; Kelly et al., 2020; Khalil et al., 2003; Khurana, 1996; Kibleur et al., 2014; Kiruki et al., 2018; Kiryluk and Sobaniec, 2013), (Klingmann et al., 2022; Kokke et al., 2008; Kokki et al., 2000; Kolaczinski et al., 2006; Kolmen et al., 1995; Kumar et al., 2012; Lagos et al., 1999; Lava et al., 2011; Leonard et al., 2014; Liacouras et al., 1993; Lin et al., 2011; Low et al., 2018; Macknin et al., 1998; Maines et al., 2020; Manford Gooch et al., 1999; Marshall et al., 2000; Matsui et al., 1996; Matsumoto et al., 2022; McIntyre and Hull, 1996; Meier et al., 2007; Melvin et al., 1997; Mettey et al., 1990; Milani et al., 2010; Mistry et al., 2018; Moniot-Ville et al., 1998; Mulla et al., 2016; Murnane et al., 2017; Nachman et al., 2014; Nahirya-Ntege et al., 2012; Nasrin et al., 2005; Nathan et al., 2006; Nezam et al., 2022; Nimrouzi et al., 2015; Nizami et al., 1996; Nuzhat et al., 2022; Osterhoudt et al., 2004; Padilla et al., 2000; Paediatric European Network for Treatment of AIDS (PENTA) (1999); Pai et al., 2023; Pajno et al., 2012; Palit et al., 2016; Paller et al., 2020; Paranthaman et al., 2009; Parshuram and McMahon, 1998; Pasipanodya et al., 2018; Patel et al., 2022; Penagini et al., 2017; Phipps and DeCuir-Whalley, 1990; Pichichero et al., 1990; Porteous et al., 1997; Powers, 1996), (Powers et al., 2000; Powers et al., 2020; Prajapati et al., 2022; Pronzini et al., 2008; Purchase et al., 2016; Purchase et al., 2019; Querin et al., 2023; Rasul and Khan, 2001; Reddington et al., 2000; Reich et al., 2000; Rendeli et al., 2006; Rittig et al., 2020; Riva et al., 1997; Rosenthal et al., 2020; Rotsaert et al., 2023; Rouse et al., 2017; Ruperto et al., 2017; Rutebemberwa et al., 2009; Saito et al., 1999; Saneian and Mostofizadeh, 2012; Savino et al., 2012; Scheuern et al., 2017; Schwarz et al., 2020; Shchelochkov et al., 2016; Silva et al., 2022; Sleath et al., 2006; Smith et al., 2013; Sondheimer et al., 1991; Splieth et al., 2000; Steans et al., 1990; Steele et al., 2002; Stevens et al., 1996; Suchismita et al., 2023; Syofyan et al., 2019; Taher et al., 2018; Tannous and Azouz, 1991; Taylor et al., 2016; Thompson et al., 2013; Thomson et al., 2015; Thornburg et al., 2011; Tiengkate et al., 2022; Todd et al., 2023; Toscani et al., 2000; Touchette et al., 1994; Tran et al., 2017; Tsouana et al., 2015; Tucker et al., 2002; Uhari et al., 1986; Unachak et al., 2002; Valayannopoulos et al., 2019), (van der Vossen et al., 2020; Van de Vijver et al., 2011; Van Dyke et al., 2002; Varma et al., 2012; Varnell et al., 2017; Varnell et al., 2021; Venables et al., 2015a; Venables et al., 2015b; Venables et al., 2018; Viprakasit et al., 2023; Voskuijl et al., 2004; Wademan et al., 2019; Warembourg et al., 2020; Warzecha et al., 2022; Wataya, 2017; Wijesekera et al., 2019; Williford et al., 2023; Xin et al., 2022; Yafout et al., 2022; Yeowell et al., 2021; Yoo et al., 2022; Young et al., 2020; Zannikos et al., 2011; Zelikovsky et al., 2011; Zhang et al., 2016). Cohen’s Kappa coefficient was κ = 0.66 for the abstract and title screening and κ = 0.76 for the full-text review, indicating substantial inter-rater reliability and agreement between the two reviewers for study inclusion.

### 3.2 Study characteristics

**FIGURE 3:**
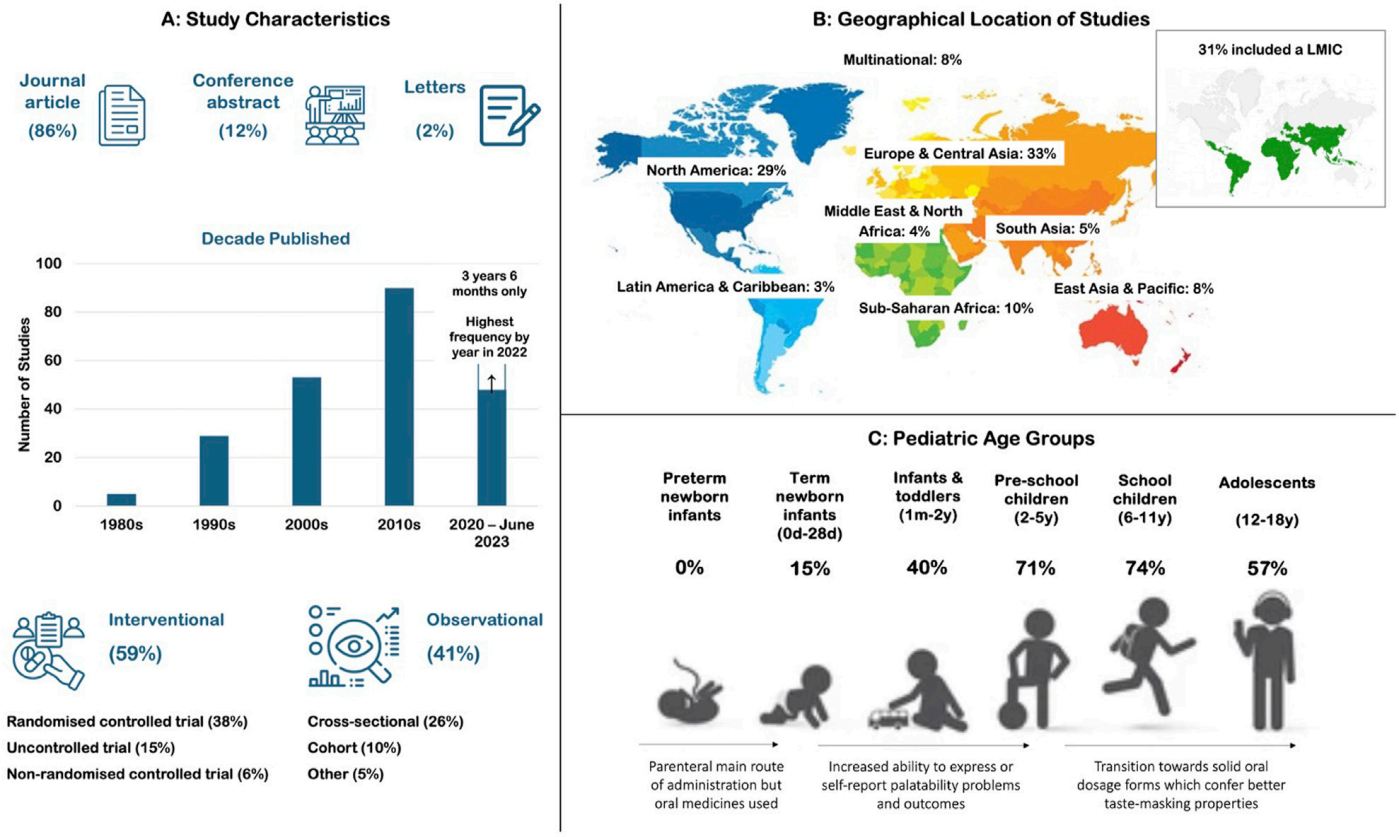
Characteristics of the 225 studies included in the review including **(A)** publication type, year, and study design; **(B)** geographical location and economic status; and **(C)** percentage of studies including each pediatric subset in the participant age range.

The characteristics of the 225 studies are summarized in Figure 3, with a complete table of results included in Supplementary Table S6. Studies were mainly journal articles (86%) and published between 1983 and 2023; however, a sharp increase in publications was observed in the last two decades coinciding with the introduction of pediatric legislative changes. A range of study designs were seen including randomized controlled trials (38%) and observational cross-sectional studies (25%). Studies were conducted across all geographical regions, although over half (62%) were conducted exclusively in high income countries in North America and Europe & Central Asia. Around a third (30%) included low- and middle-income countries (LMICs). All pediatric age groups except pre-term newborn infants were included in the studies. Over 70% included pre-school or school children, and over 50% included adolescents.

Of the studies reviewed, 47% reported palatability issues informally and linked them to outcomes, while 21% formally captured palatability issues and associated them with outcomes. Evaluation of medication palatability or acceptability was the primary objective in 15% of studies, typically for antibiotic drug products. In 5 studies, bitter or bad taste was listed as an AE but deemed to be a sensory characteristic of the API and not a systemic effect. Another 29% of studies formally captured palatability issues, but 21% did not establish a link with outcomes while 8% (n = 19) were thought to have a potential link. The remaining 2% focused solely on the general experiences of participants.

Data was captured from pediatric patients, healthy children, caregivers, and HCPs. A range of methodologies were used to formally capture palatability assessments, including rating products on scales, such as facial hedonic scales (usually with 3-, 5- or 7-point), continuous visual analogue scales (VAS) (10 cm/100 mm), and modified scales using a combination of facial hedonic and VAS. Other approaches included Likert scales, or observations of facial expressions, responses, or observed behavior following administration.

### 3.3 Overall disease area and drug classifications

**FIGURE 4:**
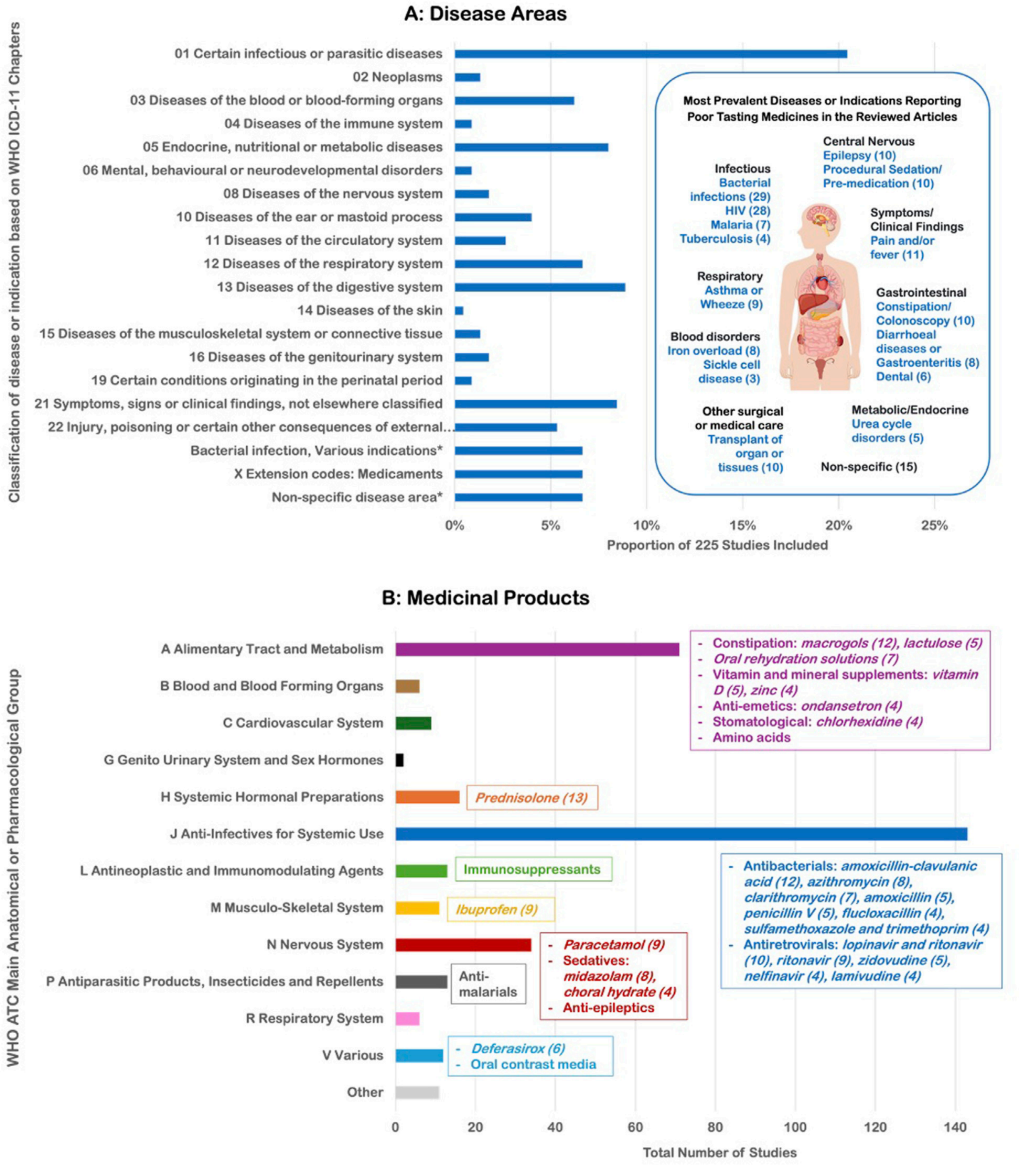
**(A)** Classification of the 77 different diseases or indications identified in the articles by WHO ICD-11 chapter and **(B)** Classification of the 156 different medicinal products reported by WHO ATC main anatomical/pharmacological group.

The impact of poor tasting medicines was observed across 77 different diseases or indications (Figure 4A). Overall, 156 different medicinal products were reported across 84% (190/225) of the reviewed articles (Figure 4B). Among these products, 93% were APIs with an ATC classification, while the remaining 7% included other unclassified interventional products used for treatment or diagnosis (e.g., herbal medicines, amino acids used in metabolic deficiency states, and products for diagnostic imaging). Poor tasting medicines were found in 12 of the 14 WHO ATC groups, highlighting that the unpleasant taste of medicines is a widespread issue across a broad range of medicinal products. The two ATC groups not covered were dermatologicals (topical preparations) and sensory organs (ophthalmic preparations). A complete list of the categorized diseases and medicinal products, along with their frequencies is included in Supplementary Tables S7, S8.

The largest proportion of conditions were notably in the infectious and parasitic diseases (20%), with studies in bacterial infections and human immunodeficiency virus (HIV) predominating. Twenty different antiviral drugs for HIV were identified as poor tasting, with lopinavir/ritonavir, ritonavir, and zidovudine the most frequently reported. Twenty-five different APIs were found across 29 studies for indications related to bacterial infections. Where palatability was formally reported (e.g., self-reported on scales), negative scores were reported for several antibiotics including amoxicillin-clavulanic acid (with 33%–61% of participants rating the taste negatively) (Block et al., 2006; Khurana, 1996; Powers et al., 2000; Steele et al., 2002), clarithromycin (36%–50%) (Manford Gooch et al., 1999; Powers, 1996; Steele et al., 2002), cefuroxime (52%–56%), sulfamethoxazole and trimethoprim (26%) (Dagan et al., 1994) and cloxacillin (mean score of 1.4 on a 10 cm VAS) (Matsui et al., 1996). Palatability reports sometimes differed between children and their caregivers. In a study of phenoxymethylpenicillin formulations, there was a weak correlation between children’s taste ratings and parents’ assessments of acceptability (Ansah et al., 2001), with some children rating the taste negatively while parents reported the medication was easily accepted at home, and *vice versa* (Bagger-Sjoback and Bondesson, 1989). In other studies, 95.7% of community pharmacists reported poor palatability of oral liquid antibiotics, especially for clarithromycin (31.5%) and flucloxacillin (28.8%) (Elgammal et al., 2022), while prescribers reported palatability issues for flucloxacillin and phenoxymethylpenicillin (Greenwood and Vallabhaneni, 2015). Over a third of caregivers reported more administration difficulties with flucloxacillin than other medicines due to taste (Batchelor et al., 2016).

The second most prevalent ICD-11 chapter encompassed symptoms or clinical findings, notably pain and fever, with the most frequent reports for ibuprofen and paracetamol, respectively. Diseases of the digestive system included constipation, where 12 studies reported poor palatability of macrogols. Prednisolone (most often in studies for asthma, wheeze or croup), oral rehydration salts (for dehydration in diarrhea or burn injury), and midazolam (for pre-procedural sedation or premedication) were the other APIs with the largest frequency of reports for poor taste.

A range of different oral dosage form types were seen across the studies. Liquid formulations included solutions, suspensions, elixirs, slurries, oral rinses, drops, sublingual sprays and topical varnishes (for dental use). Solid oral dosage form types included tablets, capsules, mini-tablets, powders, granules, chewables, dispersible and oro-dispersible preparations and lozenges. During the study screening and selection phase, some ineligible studies were found to report bitter taste sensations being perceived with non-oral dosage forms including nasal drops, eye drops, ear drops, and inhalers (Ahonen et al., 2004; Lenhard et al., 1997; Agro et al., 1998; Naimi et al., 2009); likely due to the anatomical connections between the ear, nose, and throat.

### 3.4 Impact of medication taste on patient acceptability

The impact of poor taste was most frequently seen in relation to patient acceptability, with 64% of the reviewed articles reporting rejection responses, strategies to aid administration, or effects on prescribing practices. The most frequent types of outcomes are shown in Table 1. Overall, medications used to treat HIV and bacterial infections were most commonly associated with acceptability and administration challenges. Many studies involving administration of prednisolone and midazolam also reported similar issues. Almost a quarter of studies reported incidences of children rejecting or resisting medication, with avoidance behaviors such as crying, covering the mouth, expressing discontent also described. Caregivers resorted to various approaches to overcome these challenges, ranging from positive reinforcement and rewards, to threats, physical restraint, and forced administration. These negative interventions were seen for the treatment of acute infections (Khurana, 1996) and chronic conditions such as HIV (Coetzee et al., 2015), malaria (Ewing et al., 2015), tuberculosis (Wademan et al., 2019), and metabolic disorders (Yeowell et al., 2021).

**TABLE 1:** Studies reporting outcomes related patient acceptability including rejection responses, administration strategies, and prescribing practices.

Outcomes related to patient acceptability and administration^a^	Frequency (%)
Rejection responses	
Rejecting, refusing, or resisting medication	55 (24%)
Spitting out medication	30 (13%)
Non-specific administration problem	30 (13%)
Vomiting (due to taste)	28 (12%)
Withdrawal from study	22 (10%)
Medication stopped	20 (9%)
Unwilling to take medicine in future	12 (5%)
Incomplete dosing or need for re-administration	12 (5%)
Avoidance behaviors, *e.g., crying, screaming, covering mouth, deliberately spilling medicine, running away, expressing vocal or facial discontent*	8 (4%)
Developing aversion to foods and drinks used to administer medicines	4 (2%)
Caregiver burden and interference with everyday life	3 (1%)
Strategies to aid administration	
Mixing medicines into food or drinks	56 (24%)
Manipulation of solid dosage form, *e.g., crushing tablet/opening capsule*	12 (5%)
Positive reinforcement or rewards, *e.g., praise, high-fives, providing sweets/candy*	9 (4%)
Forced administration	9 (4%)
Physical restraint or punishment	7 (3%)
Bribery or negotiation	4 (2%)
Coercion or threats	4 (2%)
Mixing with other medicines, *e.g., sweet, fruit-flavored liquids (Calpol®)*	2 (1%)
Therapeutic play, *e.g., pretending to give medicine to pet*	2 (1%)
Others approaches includes administering small amounts during the day, coating palate with peanut butter, providing an ice lolly beforehand to “freeze the taste buds”, using a straw, administering worst tasting medicines first, covert dosing, swallowing soluble tablets whole	-
Impact on prescribing practices	
Drug therapy changed	11 (5%)
Dosage form changed	7 (3%)
Sub-optimal prescribing by HCP, *e.g., use of non-first line alternative therapeutics*	5 (2%)
Administration using enteral feeding tube explicitly due to taste	3 (1%)
Compounding alternative formulations, *e.g., using vehicles to create sweetened or flavored preparations, or reformulation into capsule for taste-masking*	8 (4%)
Use of administration devices, *e.g., syringes, PillGlide spray, in-situ* coating	5 (2%)
Use of product with a “bitter blocker” *(Breeza® flavored beverage for imaging)*	1 (0.4%)

^a^ Multiple outcomes may have been reported within the same study.

The number of subjects withdrawing from studies ranged from single participants to 20% of subjects in a study with the antiretroviral darunavir/cobicistat (Dalton et al., 2019), and 42% in a study with the anti-emetic, metopimazine (Nathan et al., 2006). Taste problems also led to withdrawals over long periods of time, with 21% of patients discontinuing cholestyramine drug therapy within 1 month, and 65% within 3 years; the authors stated that the majority who discontinued the drug complained of foul taste or had difficulty with its ingestion (Liacouras et al., 1993). In 24% of studies, medicines were mixed with foods or drinks to help mitigate taste-related issues. However, when this approach was used with lopinavir/ritonavir (Giralt et al., 2019; Kekitiinwa et al., 2016), naltrexone (Kolmen et al., 1995), and magnesium salts (Mettey et al., 1990), an unintended consequence was the development of aversion to the food in which the medication was mixed. In Uganda, caregivers reported concerns about need to sweeten food with sugar or honey which can be expensive in LMIC settings (Kekitiinwa et al., 2016).

Poor tasting medicines were also found to be a barrier to optimal medical management, with prescribers reporting the need to change drug therapies or use non-first line alternative therapeutics (Bergene et al., 2019; Goode et al., 2003; Jamieson et al., 2021; Lin et al., 2011; Porteous et al., 1997). Lin et al. (2011) identified 15 antiretrovirals with poor taste, which required the need for alternative prescribing (drug change or non-first line therapy) due to patient refusal. Other approaches to overcome taste problems included use of commercial products to create a physical barrier between medications and taste buds in the mouth [e.g., PillGlide (Gaskell, 2016) and *in situ* coatings (El Edelbi et al., 2015)] and using products with excipients that are marketed to have a “bitter blocking” effect [e.g., Breeza^®^ (Dillman et al., 2018)].

### 3.5 Impact of medication taste on adherence and outcomes

Medication adherence impacts were reported in 27% (60/225) of the reviewed studies. Of these, 38 identified drug taste as a barrier to adherence, typically in chronic conditions such as organ or tissue transplant (Bullington et al., 2007; Claes et al., 2014; Pai et al., 2023; Phipps and DeCuir-Whalley, 1990; Varnell et al., 2017; Varnell et al., 2021; Wijesekera et al., 2019; Zelikovsky et al., 2011), HIV (Buchanan et al., 2012; Goode et al., 2003; Kiruki et al., 2018; Paranthaman et al., 2009; Purchase et al., 2016; Reddington et al., 2000; Tran et al., 2017), epilepsy (Gutierrez-Colina et al., 2018; Williford et al., 2023), cystic fibrosis (Christiansen et al., 2014), and inflammatory bowel diseases (Greenley et al., 2018). In a study where adherence was formally evaluated, the taste of phenoxymethylpenicillin was positively but weakly correlated with adherence, including taking 100% of the medication (coefficient 0.184, p < 0.01) and taking it at the correct time (coefficient 0.141, p < 0.01) (Cifaldi et al., 2004). Weak correlations between adherence (measured by medication return) and taste scores were also found for cefdinir and amoxicillin-clavulanic acid, with coefficients ranging from 0.1 to 0.2 (Bagger-Sjoback and Bondesson, 1989). In a study by Angwa et al. (2020), objective measures of adherence and dose accuracy were significantly better for amoxicillin dispersible tablets (DT) compared to the oral suspension (OS) (p < 0.01), with more caregivers reporting the DT as tasting better than the OS (51.5% DT vs. 38.5% OS). In another study covering various antibiotics, 21.1% of patients who did not complete their course discontinued due to poor palatability (Patel et al., 2022). In the treatment of acute childhood diarrhoea with dispersible zinc tablets, 55.8% of patients completed the 10-day treatment as advised, and those who rated the taste worse than other medicines were 51% less likely to complete the full course (RR 0.51, 95% CI 0.40–0.66) (Nasrin et al., 2005).

Only 3.5% of studies were identified where unpalatable medicines resulting in poor medication adherence had a reported impact on treatment outcomes. Two studies in HIV used the Pediatric AIDS Clinical Trials Group (PACTG) adherence module, which explicitly includes a question about taste as a drug specific reason for non-adherence (Davies et al., 2008; Van Dyke et al., 2002). Davies et al. (2008) found that taste was the most common barrier to adherence reported by over 20% of respondents, with 21% of participants being non-adherent, defined as an annual medication return <90%. Among adherent children, 78% had an undetectable viral load, compared to 25% of non-adherent children, such that adherent children were over 10 times more likely to achieve viral suppression (OR 10.3 95% CI: 1.92–55.7; p = 0.005) (Davies et al., 2008). Non-adherence was linked to the poor taste of ritonavir. Another study using the same approach found that 30% of children were non-adherent, with 5% missing all doses (Van Dyke et al., 2002). Among those that were non-adherent, 44% had an undetectable RNA viral load compared to 64% who were adherent (p = 0.07). Non-adherence was associated with the taste of ritonavir and nelfinavir. In a chronic hepatitis C virus (HCV) infection study, sustained viral suppression was not achieved in a 4-year-old patient who discontinued treatment due to the poor taste of sofosbuvir (Rosenthal et al., 2020). The drug, formulated as granules in a capsule, was intended to be sprinkled on a spoonful of non-acidic soft food; however, acidic food was mistakenly used, likely causing the granule coating to break and leading to the unpleasant taste of the drug being perceived.

In epilepsy, changes to more palatable formulations of valproic acid were associated with an improved pharmacokinetic profile, positive changes in the EEG trace, and reduction in frequency of epileptic seizures (Cloyd et al., 1992; Kiryluk and Sobaniec, 2013). Gutierrez-Colina et al. (2018) captured taste difficulties within the Pediatric Epilepsy Medication Self-Management Questionnaire (PEMSQ). For children aged 2–5 years, medication taste was the most significant predictor of seizure control and health-related quality of life (HRQOL), while in adolescents aged 13–17 years, disliking the taste of medication was the most important predictor of non-adherence, and second most important predictor of seize control and HRQOL (Gutierrez-Colina et al., 2018). In a study of urea cycle disorder patients, the existing sodium phenylbutyrate (NaPB) product was deemed unacceptable by all 25 patients, with 4 finding it impossible to take. Two patients required NaPB to be reformulated into capsules, 4 needed it to be administered via a nasogastric tube (NGT), and 1 required gastrostomy administration. After switching to a new coated oral pellet formulation (Pheburane) acceptability scores improved, and in 10 patients, the number of hyperammonemia episodes dropped from 20 over 6 months on NaPB to zero over 3–11 months of treatment with Pheburane (Kibleur et al., 2014). The authors observed an inverse relationship between acceptability and bitterness, though this correlation was not formally tested. Unachak et al. (2002) described a case report in neonatal hypomagnesaemia, where poor adherence to magnesium sulfate due to its bitter taste, led to persistent hypomagnesemia, hypocalcemia, and convulsions. Changing to magnesium oxide led to improved compliance and resolution of hypomagnesemia though intermittent symptoms, such as convulsions reappeared due to poor regular adherence with the treatment.

### 3.6 Other findings related to medication taste

Taste was found to influence both beliefs and treatment preferences in several studies. Over a quarter (26.4%) of Indonesian children aged 10–14 years believed that the taste of a medicine, whether sweet or bitter, impacted its efficacy (Syofyan et al., 2019) while caregivers in Uganda associated bitter taste with the authenticity of medicines, with one participant remarking, “if it wasn’t bitter, it would be fake” (Rutebemberwa et al., 2009). In a discrete choice experiment on pediatric tuberculosis preventive treatment preferences, taste was the most significant factor influencing choice among children, caregivers, and healthcare providers in Eswatini (Hirsch-Moverman et al., 2021). Participants were more than three times likely to choose an alternative treatment if the treatment option presented was described as bitter (OR = 3.51, 95% CI: 2.81–4.38), with this preference for non-bitter formulations outweighing other factors such as treatment duration, dose frequency, pill size, and cost (Hirsch-Moverman et al., 2021).

## 4 Discussion

### 4.1 Summary of evidence

This scoping review has highlighted the significant adverse impact of poor-tasting pediatric medicines on patient and stakeholder experiences. The strongest evidence linked poor taste to challenges with medication administration and acceptability, followed by reports that it is a notable barrier to medication adherence, and to a lesser extent, the resulting negative impact on treatment outcomes. The issue of unpleasant drug taste affects a wide range of disease areas and APIs, spanning over 70 different acute and chronic indications and more than 150 treatment products. Poor palatability was experienced with a range of solid and liquid oral dosage forms, and extended to other modes of administration that lead to drug exposure in the ear, nose, and throat. This problem is prevalent globally, impacting almost the entire pediatric population, from newborns to adolescents. The negative effects of poor-tasting medicines have been reported by children, their parents or caregivers, as well as a variety of HCPs, including doctors, nurses, and pharmacists. These issues have been observed in both experimental studies and real-world settings.

### 4.2 Impact of poor-tasting medicines in pediatric care

Poor-tasting medicines identified in this review include several of the most commonly prescribed treatments for pediatric patients. Analysis of global pharmacy sales data from 75 countries (including 49% LMICs) for 47 oral medicines on the WHO Essential Medicines List for Children (EMLc) revealed the top 10 selling medicines (Tsai et al., 2022). The data focused on child-appropriate formulations, defined as liquid syrups, granules, powders/dry syrups, and dispersible tablets with a standard unit (SU) representing a single dose (one tablet, capsule, ampoule, vial, or 5 mL of liquid). Paracetamol topped the list with 31.1% of total sales, amounting to 11,090 million standard units (SU), followed by amoxicillin (11.8%, 4,206 million SU), ibuprofen (10.8%, 3,870 million SU), and amoxicillin-clavulanic acid (9.9%, 3,549 million SU). Other top-selling medicines included prednisolone (8.4%), cefixime (5.2%), valproic acid (3.0%), sulfamethoxazole-trimethoprim (2.5%), lamotrigine (2.3%), and azithromycin (2.1%). Nine of these top ten medicines were identified in this review, with the exception of lamotrigine. The failure to administer poor-tasting pediatric medicines results in billions of missed doses each year. In a UK study by Mistry et al. (Mistry et al. 2018), researchers observed that refusal, spitting out, or vomiting of liquid medicines occurred most frequently with the top 5 highest selling medicines: in 1% of those taking paracetamol, 13% taking amoxicillin, 2% taking ibuprofen, 6% taking amoxicillin-clavulanic acid, and 9% taking prednisolone. Extrapolating these percentages to the global sales data (Tsai et al., 2022), the number of missed doses globally could reach over 110 million for paracetamol, 546 million for amoxicillin, 77 million for ibuprofen, 212 million for amoxicillin-clavulanic acid, and 268 million for prednisolone.

The issue of poor taste has been recognized in several other studies. In a large survey of nearly 700 European children, the most common reason for difficulty in taking medicines was a dislike of the taste, as reported by 63.7% of respondents (Nordenmalm et al., 2019). Taste issues were reported with 35% (188/542) of all prescribed oral formulations in cross-sectional UK study, with children experiencing a palatability issue to be almost four times more likely to refuse taking the medication compared to those who did not (Venables et al., 2015a). At least 50% of children prescribed ranitidine, prednisolone, trimethoprim, lactulose, macrogol, co-trimoxazole, sodium valproate, levetiracetam, phenoxymethylpenicillin, and ibuprofen reported experiencing taste-related issues, further highlighting the widespread and pervasive nature of this problem (Venables et al., 2015a).

Medicines for the treatment of bacterial infections and HIV were frequently reported to have taste issues, which were linked to adherence challenges and treatment outcomes. In a global survey of HCPs, oral liquid forms of lopinavir/ritonavir, amoxicillin and clavulanic acid, and cefuroxime were most frequently reported as problematic, including for acceptability and palatability issues (Barbieri et al., 2023). Infections remain one of the leading worldwide causes of mortality in children under five (Perin et al., 2022). A narrative review by Baguley et al. (Baguley et al., 2012) discussed how palatability can impact adherence to antibiotics, highlighting flucloxacillin suspension as a particularly challenge drug, consistent with the findings in this review. The authors emphasized the need for doctors to be more aware of the importance of taste when prescribing medications for pediatric patients. Indeed, in a study where HCPs assessed the palatability of twenty-four liquid anti-infectives, participants experienced firsthand how certain formulations were more palatable than others, and many subsequently adjusted their prescribing or counselling practices (Gee and Hagemann, 2007). Failure to complete antibiotic treatment or not taking them as prescribed can lead to prolonged illness, increased risk of complications, and contribute to wider public health threats, including the spread of infectious diseases.

In 2023, an estimated 1.4 million children (0–14 years old) were living with HIV; only around 48% of children living with HIV had suppressed viral loads and there were 76,000 deaths from HIV-related causes in this population (World Health Organization, 2024). The poor taste of ritonavir (alone and in combination with lopinavir) was reported in nineteen studies in the review. These drugs have shown to activate the TAS2R bitter receptors which mediate the perception of this taste sensation in the mouth (Chen et al., 2023). Perceiving the bitter taste of drugs is a key factor contributing to poor palatability. In the accompanying article in this series, between 18%–60% of caregivers reported that their child always or regularly refused medication due to bitter taste (El-Sahn et al., 2025), while over 80% of healthcare providers agreed that bitter taste impacts adherence to both short-term and long-term medications.

Acceptability and adherence are complex, multidimensional phenomena that can be impacted by a myriad of factors. WHO describes five interacting dimensions that can affect adherence: therapy–related factors; patient–related factors; condition–related factors; social and economic factors; and healthcare team and system-related factors (World Health Organization, 2003). These dynamics can also influence patient acceptability of medicines. Caregiver-related factors can also be considered another important dimension in pediatrics, since parents and carers play an important role in medicines administration in this population. A range of administration difficulties and associated coping mechanisms used by caregivers were identified in the review, varying from supportive to detrimental. This highlights the significant burden and psycho-social impact these challenges pose for pediatric patients and their families. Similar to the findings of this review, a qualitative study by Bergene et al. (2017) categorized three strategies parents use to administer oral medicine to children: open administration (involving the child or altering the taste), hidden administration (camouflaging in food or distractions), and forced administration (using restraint), with varying parental attitudes towards the use of force. The use of strategies such as masking the bitter taste of medicine with food or drink, giving rewards or the use of force were reported by caregivers in Sub-Saharan Africa and the United States in the second part of this series (El-Sahn et al., 2025). This emphasizes the need for targeted supportive measures to alleviate these difficulties for all stakeholders.

### 4.3 Regulatory landscape and equitable global access

The U.S. and EU introduced legislative and regulatory reforms over a decade ago to incentivize and mandate the development of pediatric medicines (Penkov et al., 2017). These advancements have shown a positive impact on the authorization of new medicinal products and pharmaceutical formulations; however, efforts to develop off-patent medicines have been notably less successful, and universal access to these new medicines is not assured (European Medicines Agency, 2017). A limitation of this review was the inability to analyze poor taste at the product-specific level. Notably, other reviews have highlighted that significant variations in palatability exist between different formulations of the same API, particularly when comparing originator versus generic products (Tuleu et al., 2021). There is no regulatory requirement for palatability testing of generic drugs, leading to potential issues with unpalatable formulations entering the market. The findings of this review are important, given the unequal access to various medicinal products worldwide. There remains a critical global need for age-appropriate and accessible medicines, especially in LMICs (Barbieri et al., 2023; Chen et al., 2021). This is pertinent as the proportion of the pediatric population rises in the least developed countries, representing 48% of sub-Saharan Africa versus 21% in North America, and Europe/Central Asia (United Nations, 2022).

The current review was based on an initial screen of titles and abstracts due to the large volume of articles identified, which may have led to the omission of other relevant studies where these outcomes were not considered the main findings. However, despite the widespread impact of poor tasting medicines found in this review, the prevalence of this issue is likely underreported due to several factors. Historically, the taste or palatability of medicines has not been evaluated as a primary or even secondary outcome in clinical trials. Limited data exists on the direct impact of poor taste on patient outcomes, not due to an absence of effect, but because this problem has not been systematically captured or analyzed in research studies to date. Studies on medication adherence are often disease specific and typically focus on patients’ beliefs, knowledge, and behaviors in relation to medicines use (Nguyen et al., 2014). Kardas et al. identified over 700 individual factor items associated with non-adherence to chronic therapies in patients of all ages, with only 3/51 reviews in their analysis mentioning poor taste of medication as a therapy-related factor affecting adherence (Kardas et al., 2013). Incorporating medication taste as a variable in adherence questionnaires and assessment tools is essential to help systematically evaluate its impact on medication adherence. Therapeutic outcomes are typically considered in relation to clinical pharmacology and drug efficacy and even less likely analyzed in relation to medication taste. Notably, regulatory agencies now require the evaluation of patient acceptability as an integral part of the pharmaceutical and clinical development of pediatric medicines, a shift that is expected to help address these evidence gaps and further elucidate the impact of palatability on outcomes (European Medicines Agency, 2013; Food and Drug Administration, 2018).

### 4.4 Taste masking pediatric formulations

The palatability of a medicines is determined by the characteristics of the API, the excipients used in the formulation, and the finished medicinal product (European Medicines Agency, 2013). Existing reviews provide a detailed exploration of taste-masking strategies and technologies (Hu et al., 2023; Sanjay et al., 2025). These approaches typically involve either blocking taste transmission pathways or preventing drug release in the oral cavity. Examples range from the inclusion of sweeteners and flavors or coating solid oral dosage forms with polymer barriers, to more advanced techniques such as microencapsulation, nanotechnology, ion-exchange resin complexation, and use of bitterness inhibitors. When selecting taste-masking strategies, the goal is to enhance palatability without compromising other critical aspects of the drug product, such as stability, bioavailability, and safety.

The scoping review identified examples where modifications to formulation factors improved the palatability and acceptability of pediatric medicines. A film-coated tablet formulation of deferasirox improved palatability compared to the dispersible tablet by eliminating taste and aftertaste issues (Taher et al., 2018). In this clinical study, patient-reported outcomes were captured using specifically designed questionnaires assessing palatability and satisfaction. A 4-in-1 fixed-dose combination of abacavir/lamivudine/lopinavir/ritonavir was specifically designed for infants and young children with HIV; this strawberry flavored granule in capsule formulation can be taken with liquids or semi-solid food (Rotsaert et al., 2023). In a qualitative study, caregivers generally reported that the formulation had an appealing taste compared with previously used formulations; though, reports of spitting attributable to the new taste were nevertheless also reported (Rotsaert et al., 2023). These recently published studies demonstrate the importance of considering taste as a critical factor when formulating pediatric medicines and the benefits of evaluating this aspect with patients and caregivers. Further robust studies are needed to close the evidence gap, provide insights into the appropriate methods to capture palatability, and confirm that better-tasting medicines are indeed benefiting patients.

### 4.5 Other limitations

Some limitations of this review have been addressed, including the inability to analyze poor taste of medicines at the product-specific level, and the potential exclusion of relevant articles during the title and abstract screening. Assessment of methodological limitations or risk of bias of the evidence was not conducted as part of the appraisal process, though this acceptable within the remit of a scoping review. The review did not assess the significance of the number of participants reporting taste-related issues. Instead, any mention of palatability or taste issues, including single instances, was included, limiting the ability to evaluate the degree of taste problems. Current regulatory guidelines do not specify a threshold for the proportion of individuals who must find a medicine palatable or acceptable, which restricts the ability to make meaningful comparisons regarding the extent of taste-related challenges across different medications.

### 4.6 Conclusion and future directions

This scoping review highlights the significant impact of poor-tasting pediatric medicines on medication acceptability, adherence, and ultimately treatment outcomes. Despite the recognition of this challenge, the current body of evidence remains incomplete with regards to the true extent of the problem.

There is a need for more targeted research on palatability and more comprehensive guidance for its evaluation of both new and generic pediatric formulations. Regulatory requirements are playing a pivotal role in driving improvements in palatability assessments; however, the current absence of mandatory assessments for generic formulations creates a gap in ensuring that these medicines meet the same acceptability standards as branded counterparts. Bridging this gap will also support more equitable access to better formulations across different populations, especially in LMICs.

The economic burden of medication wastage due to poor taste is substantial, with millions of missed doses annually. Addressing taste-related challenges could thus have significant cost-saving implications. Raising awareness among HCPs about the impact of medication taste is essential, as these stakeholders are in a key position to engage patients and caregivers and offer education, support, and effective strategies to help overcome administration challenges. Incorporating taste and formulation-related factors into wider research areas, including adherence measures and longitudinal studies, will provide a deeper understanding of the long-term effects of poor-tasting medicines on children’s health and development. This research could also explore broader impacts, such as how medication challenges may influence social behaviors related to healthcare.

Research and development into both existing and innovative taste-masking strategies is critical to help resolve this problem with poor-tasting medicines. The concept of a taste-blocker product is explored in the accompanying article in this series (El-Sahn et al., 2025), as a universal solution that could support the administration of different medications, and be helpful even when taste problems occur in only a few patients. Additionally, developing formulation technologies that allow for more widespread application of taste-masking techniques, without compromising product stability or therapeutic efficacy, would be a significant step forward. Addressing the problem of poor taste is critical to improving the overall experience of taking medicines for children and their caregivers worldwide.

## Data Availability

The original contributions presented in the study are included in the article/Supplementary Material, further inquiries can be directed to the corresponding author.
