# Supplementary material for: Poor-tasting pediatric medicines: Part 1. A scoping review of their impact on patient acceptability, medication adherence, and treatment outcomes
Source: Front Drug Deliv. 2025 Apr 22;5:1553286. doi: 10.3389/fddev.2025.1553286 (PMC12360445; doi:10.3389/fddev.2025.1553286)
Supplement: Supplementary file 1 [file DataSheet1.pdf]

Poor-tasting pediatric medicines: Part 1. A scoping review of their impact on patient acceptability, medication adherence, and treatment outcomes

Supplementary Material

1 Supplementary Tables

Table S1. PRISMA-ScR Checklist with items and criteria recommended for transparent and comprehensive reporting of scoping reviews.

SECTION	ITEM	PRISMA-ScR CHECKLIST ITEM	REPORTED
**TITLE**			
Title	1	Identify the report as a scoping review.	Title
**ABSTRACT**			
Structured summary	2	Provide a structured summary that includes (as applicable): background, objectives, eligibility criteria, sources of evidence, charting methods, results, and conclusions that relate to the review questions and objectives.	Abstract
**INTRODUCTION**			
Rationale	3	Describe the rationale for the review in the context of what is already known. Explain why the review questions/objectives lend themselves to a scoping review approach.	Introduction
Objectives	4	Provide an explicit statement of the questions and objectives being addressed with reference to their key elements (e.g., population or participants, concepts, and context) or other relevant key elements used to conceptualize the review questions and/or objectives.	Introduction
**METHODS**			
Protocol and registration	5	Indicate whether a review protocol exists; state if and where it can be accessed (e.g., a Web address); and if available, provide registration information, including the registration number.	Methods; registration n/a for scoping review
Eligibility criteria	6	Specify characteristics of the sources of evidence used as eligibility criteria (e.g., years considered, language, and publication status), and provide a rationale.	Methods 2.1
Information sources	7	Describe all information sources in the search (e.g., databases with dates of coverage and contact with authors to identify additional sources), as well as the date the most recent search was executed.	Methods 2.2 & Supplementary Table S2
Search	8	Present the full electronic search strategy for at least 1 database, including any limits used, such that it could be repeated.	Methods 2.2 & Supplementary Table S3
Selection of sources of evidence	9	State the process for selecting sources of evidence (i.e., screening and eligibility) included in the scoping review.	Methods 2.3

SECTION	ITEM	PRISMA-ScR CHECKLIST ITEM	REPORTED
Data charting process	10	Describe the methods of charting data from the included sources of evidence (e.g., calibrated forms or forms that have been tested by the team before their use, and whether data charting was done independently or in duplicate) and any processes for obtaining and confirming data from investigators.	Methods 2.4
Data items	11	List and define all variables for which data were sought and any assumptions and simplifications made.	Methods 2.4 & Supplementary Table S4
Critical appraisal of individual sources of evidence	12	If done, provide a rationale for conducting a critical appraisal of included sources of evidence; describe the methods used and how this information was used in any data synthesis (if appropriate).	Methods 2.5 & Supplementary Table S5
Synthesis of results	13	Describe the methods of handling and summarizing the data that were charted.	Methods 2.5
**RESULTS**			
Selection of sources of evidence	14	Give numbers of sources of evidence screened, assessed for eligibility, and included in the review, with reasons for exclusions at each stage, ideally using a flow diagram.	Results 3.1 & Figure 2
Characteristics of sources of evidence	15	For each source of evidence, present characteristics for which data were charted and provide the citations.	Results 3.2, Figure 3 & Supplementary Table S6
Critical appraisal within sources of evidence	16	If done, present data on critical appraisal of included sources of evidence (see item 12).	N/A
Results of individual sources of evidence	17	For each included source of evidence, present the relevant data that were charted that relate to the review questions and objectives.	Results 3.3 – 3.6 & Supplementary Tables S7 & S8
Synthesis of results	18	Summarize and/or present the charting results as they relate to the review questions and objectives.	Results 3.3 – 3.6
**DISCUSSION**			
Summary of evidence	19	Summarize the main results (including an overview of concepts, themes, and types of evidence available), link to the review questions and objectives, and consider the relevance to key groups.	Discussion 4.1 – 4.4
Limitations	20	Discuss the limitations of the scoping review process.	Discussion 4.5
Conclusions	21	Provide a general interpretation of the results with respect to the review questions and objectives, as well as potential implications and/or next steps.	Discussion 4.6
**FUNDING**			
Funding	22	Describe sources of funding for the included sources of evidence, as well as sources of funding for the scoping review. Describe the role of the funders of the scoping review.	Funding

Table S2 Keywords and index search terms used based on Population-Exposure-Outcome (PEO) framework

PEO Framework Item	Criteria	Keywords/Search Terms
Population	Children 0-18 years	Pediatrics<br>Infant, Child, Adolescent
Exposure	Administration of an oral unpalatable medicine	Palatability, Taste, Smell, Texture, Mouthfeel, Bitter, Aversive Medication, Drug Therapy, Formulation, Dosage form
Outcome	Non-acceptance<br>Non-adherence<br>Treatment Outcomes	Acceptability<br>Adherence, Compliance, Concordance<br>Treatment Outcomes

Table S3 Full database search strategies

Search Query		Results
**Medline via PubMed**		
Search: ((((((pediatric) OR (child)) OR (infant)) OR (adolescent)) AND (((((((palatab*) OR (taste)) OR (smell)) OR (texture)) OR (mouthfeel)) OR (bitter)) OR (avers*))) AND ((((drug therapy) OR (drug formulation)) OR (drug dosage form)) OR (medication))) AND ((((adherence) OR (medication compliance)) OR (accept*)) OR (treatment outcome)) Filters: English		1563
**Translations**<br>**pediatric:** "paediatrics"[All Fields] OR "pediatrics"[MeSH Terms] OR "pediatrics"[All Fields] OR "paediatric"[All Fields] OR "pediatric"[All Fields]<br>**child:** "child"[MeSH Terms] OR "child"[All Fields] OR "children"[All Fields] OR "child's"[All Fields] OR "children's"[All Fields] OR "childrens"[All Fields] OR "childs"[All Fields]<br>**infant:** "infant"[MeSH Terms] OR "infant"[All Fields] OR "infants"[All Fields] OR "infant's"[All Fields]<br>**adolescent:** "adolescences"[All Fields] OR "adolescency"[All Fields] OR "adolescent"[MeSH Terms] OR "adolescent"[All Fields] OR "adolescence"[All Fields] OR "adolescents"[All Fields] OR "adolescent's"[All Fields]<br>**taste:** "taste"[MeSH Terms] OR "taste"[All Fields] OR "tastes"[All Fields] OR "tasting"[All Fields] OR "tasted"[All Fields] OR "tasteful"[All Fields] OR "tastings"[All Fields]<br>**smell:** "smell"[MeSH Terms] OR "smell"[All Fields] OR "smelled"[All Fields] OR "smelling"[All Fields] OR "smells"[All Fields]<br>**texture:** "textural"[All Fields] OR "texturally"[All Fields] OR "texture"[All Fields] OR "texture's"[All Fields] OR "textured"[All Fields] OR "textures"[All Fields] OR "texturing"[All Fields] OR "texturization"[All Fields] OR "texturize"[All Fields] OR "texturized"[All Fields] OR "texturizing"[All Fields]<br>**bitter:** "aversive agents"[Pharmacological Action] OR "aversive agents"[MeSH Terms] OR ("aversive"[All Fields] AND "agents"[All Fields]) OR "aversive agents"[All Fields] OR "bitterants"[All Fields] OR "bitter"[All Fields] OR "bitterant"[All Fields] OR "bitterness"[All Fields] OR "bitters"[All Fields]<br>**drug therapy:** "drug therapy"[Subheading] OR ("drug"[All Fields] AND "therapy"[All Fields]) OR "drug therapy"[All Fields] OR "drug therapy"[MeSH Terms] OR ("drug"[All Fields] AND "therapy"[All Fields])<br>**drug formulation:** "drug compounding"[MeSH Terms] OR ("drug"[All Fields] AND "compounding"[All Fields]) OR "drug compounding"[All Fields] OR ("drug"[All Fields] AND "formulation"[All Fields]) OR "drug formulation"[All Fields]<br>**dosage form:** "dosage forms"[MeSH Terms] OR ("dosage"[All Fields] AND "forms"[All Fields]) OR "dosage forms"[All Fields] OR ("dosage"[All Fields] AND "form"[All Fields]) OR "dosage form"[All Fields]<br>**medication:** "medic"[All Fields] OR "medical"[All Fields] OR "medicalization"[MeSH Terms] OR "medicalization"[All Fields] OR "medicalizations"[All Fields] OR "medicalize"[All Fields] OR "medicalized"[All Fields] OR "medicalizes"[All Fields] OR "medicalizing"[All Fields] OR "medically"[All Fields] OR "medicals"[All Fields] OR "medicated"[All Fields] OR "medication's"[All Fields] OR "medics"[All Fields] OR "pharmaceutical preparations"[MeSH Terms] OR ("pharmaceutical"[All Fields] AND "preparations"[All Fields]) OR "pharmaceutical preparations"[All Fields] OR "medication"[All Fields] OR "medications"[All Fields]<br>**adherence:** "adherance"[All Fields] OR "adhere"[All Fields] OR "adhered"[All Fields] OR "adherence"[All Fields] OR "adherences"[All Fields] OR "adherent"[All Fields] OR "adherents"[All Fields] OR "adherer"[All Fields] OR "adherers"[All Fields] OR "adheres"[All Fields] OR "adhering"[All Fields]<br>**medication compliance:** "medication adherence"[MeSH Terms] OR ("medication"[All Fields] AND "adherence"[All Fields]) OR "medication adherence"[All Fields] OR ("medication"[All Fields] AND "compliance"[All Fields]) OR "medication compliance"[All Fields]<br>**treatment outcome:** "treatment outcome"[MeSH Terms] OR ("treatment"[All Fields] AND "outcome"[All Fields]) OR "treatment outcome"[All Fields]		
**EMBASE via OVID**		
1	(Pediatric or Child or Infant or Adolescent).af.	4445348
2	(Palatab* or Taste or Smell or Texture or Mouthfeel or Bitter or Avers*).af.	178677
3	(Drug therapy or Drug formulation or Drug dosage form or Medication).af.	5729350
4	(Adherence or Medication compliance or Accept* or Treatment outcome).af.	1985133
5	1 and 2 and 3 and 4	1076

6	limit 5 to English language		1018
**CINAHL via EBSCO**			
S5	S1 AND S2 AND S3 AND S4		326
S4	TX adherence OR TX medication compliance OR TX accept* OR TX treatment outcome	Expanders - Apply equivalent subjects	671,011
S3	TX drug therapy OR TX drug formulation OR TX drug dosage form OR TX medication	Narrow by Language: - English	2,819,393
S2	TX palatab* OR TX taste OR TX smell OR TX texture OR TX mouthfeel OR TX bitter OR TX avers*	Search modes - Boolean/Phrase	24,051
S1	TX pediatric OR TX child OR TX infant OR TX adolescent		1,624,310

Table S4 Data extraction template used within the Covidence software

**General Information**	
1	Title, Year, Author(s) – automatically captured in
2	Country (If specified in text) OR Authors Country [Open Text]
**Study Details**	
3	Overall Category [Multiple Tick Box]<br>☐ Taste problem formally captured and directly linked to adherence or treatment outcome(s)<br>☐ Taste problem formally captured and linked to acceptability/administration outcome(s)<br>☐ Taste problem formally captured with potential (but not direct) link to outcome(s)<br>☐ Taste problem formally captured but no impact on outcomes<br>☐ Palatability not formally evaluated but linked to adherence or treatment outcome(s)<br>☐ Palatability not formally evaluated but linked to acceptability/administration outcome(s)<br>☐ Taste problem reported as adverse event (AE)<br>☐ General statement about palatability<br>☐ Palatability reported as barrier to adherence<br>☐ Patient / caregiver experiences (general)<br>☐ HCP experiences (general)<br>☐ Specific palatability/acceptability study with children<br>☐ Other [Open Text]<br>Additional notes about overall category [Open Text]
4	Was the palatability of a product/API formally evaluated? [Single Tick Box]<br>○ Yes - user questionnaire/survey, observation<br>○ Yes - taste problem reported as an AE<br>○ No but authors provide a general statement about palatability<br>○ No<br>○ Not applicable e.g. study not API or product specific<br>○ Other [Open Text]
5	Was palatability specifically linked to an outcome by the authors? [Multiple Tick Box]<br>☐ Yes - Acceptability/Administration<br>☐ Yes - Adherence<br>☐ Yes - Clinical Treatment Outcome<br>☐ No<br>☐ Other [Open Text]
6	Study Design [Multiple Tick Box]<br>☐ Interventional - uncontrolled (no comparison between interventions)<br>☐ Interventional - randomised control trial<br>☐ Interventional - non-randomised control trial<br>☐ Observational - Cohort Prospective<br>☐ Observational - Cohort Retrospective<br>☐ Observational - Case-Control Retrospective<br>☐ Observational - Cross Sectional<br>☐ Observational - Non-comparative (case series, case study)<br>☐ Other [Open Text]<br>Additional notes about overall category [Open Text]
7	Methods [Multiple Tick Box]<br>☐ Questionnaire/Survey<br>☐ Observation

	☐ Semi-structured Interview Focus Group<br>☐ Chart Review<br>☐ Other [Open Text]
8	Study Respondent [Multiple Tick Box]<br>☐ Child<br>☐ Primary adult caregiver<br>☐ Healthcare provider<br>☐ Author report<br>☐ Other [Open Text]
**Pediatric Population**	
9	Overall Category [Open Text]<br>Age – Mean<br>Age - Median<br>Age - Range<br>Sex - Female %<br>Sex - Male %<br>Total number of participants<br>Healthy Volunteers?<br>Any additional notes about study participants [Open Text]
**Medicinal Product & Disease**	
10	Therapeutic area (e.g. HIV, malaria, TB) [Open Text]
11	Drug, Dosage form, Additional Product Information [Table]
**Formal Palatability/Acceptability Evaluation**	
12	Outcome type [Single Tick Box]<br>○ Primary<br>○ Secondary<br>○ Other [Open Text]
13	Methods (E.g. scale - VAS, Hedonic, Likert) [Open Text]
14	Respondent for palatability/acceptability assessment [Multiple Tick Box]<br>☐ Child<br>☐ Caregiver<br>☐ Healthcare provider<br>☐ Other
15	Results [Open Text]
**General Palatability/Acceptability Reports**	
16	Methods (e.g. questionnaire, interview, focus group) [Open Text]
17	Results [Open Text]<br>Any additional notes about palatability [Open Text]
**Adherence**	
18	Methods (e.g. e.g. self-reported adherence, pill returns) [Open Text]
19	Results [Open Text]<br>Any additional notes about adherence [Open Text]
**Clinical/Treatment Outcomes**	
20	Methods (e.g. clinical cure, viral suppression) [Open Text]
21	Results [Open Text]<br>Any additional notes about adherence [Open Text]

Acceptability/Administration Outcomes	
22	Method of assessment [Open Text]
23	Outcomes [Multiple Tick Box]<br>☐ Spitting out<br>☐ Vomiting<br>☐ Rejecting/resistance<br>☐ Refusal<br>☐ Withdrawal from study<br>☐ Medication stopped<br>☐ Drug therapy changed<br>☐ Dosage form changed<br>☐ Unspecified administration problem<br>☐ Other [Open Text]
24	Strategies to aid administration [Multiple Tick Box]<br>☐ Mixing with food<br>☐ Mixing with drinks<br>☐ Manipulation (crushing tablet/opening capsule)<br>☐ Administration device<br>☐ Positive reinforcement e.g. praise, high-fives<br>☐ Reward<br>☐ Negotiation<br>☐ Bribery<br>☐ Coercion / threats<br>☐ Physical restraint<br>☐ Forced administration<br>☐ Other [Open Text]<br>Additional notes regarding outcomes [Open Text]
**Other Study Information**	
25	Other Notes [Open Text]

Table S5 Process of study appraisal for inclusion and analysis in relation to palatability and outcome reports

Report of Unpalatable/ Poor Tasting Medicine		Impact on Patient Acceptability	Impact on Medication Adherence	Impact on Therapeutic Outcomes	Other
**Inclusion**					
• Formal report: Palatability or taste rated by participant or caregiver<br>• Informal report: Authors report a palatability problem <u>and</u> impact, but no formal methodology described<br>AND<br>• Any degree of negative response reported by any single participant	⟼	• Rejection response (e.g. spitting out, resistance,).<br>• Practical strategies to aid administration (e.g. mixing with food/drink, need for positive reinforcement or forced administration).<br>• Impact on prescribing (e.g. drug therapy changed, dose form changed, clinician prescribing decisions).	• Adherence measure described (e.g. pill count, self-reported missed doses) and sub-optimal adherence linked to palatability.<br>• Taste problem reported as barrier to adherence (e.g. using measurement tool or during semi-structured interview/focus group discussion).	• Authors discuss therapeutic outcomes in relation to palatability (correlation or inferred impact stated).	• Patient or caregivers' beliefs or opinions about palatability in relation to medicines use (e.g. perceived association with drug efficacy).
**Exclusion**					
• Product preference in relation to palatability (e.g. product A tasted better than product B).<br>• All palatability ratings positive.		• Authors simply state that palatability can influence acceptability or administration.	• Authors simply state that palatability can impact adherence, but no formal data captured.	• Clinical outcomes reported but findings not linked or discussed in relation to taste.	

Table S6 Characteristics of the 225 studies included in the scoping review

Study Characteristics (N=225)	N (%)
**Publication Year**	
1980 - 1989	5 (2%)
1990 – 1999	29 (13%)
2000 – 2009	53 (24%)
2010 - 2019	90 (40%)
2020 – June 2023	48 (21%)
**Publication Type**	
Journal article	193 (86%)
Conference proceeding	28 (12%)
Letter	4 (2%)
**Study Design**	
Interventional - Randomized controlled trial	86 (38%)
Interventional - Uncontrolled trial	34 (15%)
Interventional - Non-randomized control trial	14 (6%)
Observational - Cross Sectional	57 (25%)
Observational - Other (e.g. cohort, case study)	34 (15%)
**Geographical Region**	
Europe & Central Asia	74 (33%)
North America	65 (29%)
Sub-Saharan Africa	23 (10%)
East Asia & Pacific	19 (8%)
Multinational	18 (8%
South Asia	12 (5%)
Middle East & North Africa	8 (4%)
Latin America & Caribbean	6 (3%)
**Research in LMICs**	
Yes – study site includes LMIC	68 (30%)
No - high income country only	157 (70%)
**Pediatric Age Group (if included in study age range)**	
Preterm newborn infants	0 (0%)
Term newborn infants (0 to 27 days)	33 (15%)
Infants and toddlers (28 days to 23 months)	91 (40%)
Pre-school children (2-5 years)	159 (71%)
School children (6-11 years)	167 (74%)

Adolescents (12-18 years)	128 (57%)
Age range not specified	20 (9%)
**Overall study classification**	
Palatability issue informally reported and linked with outcome(s)	105 (47%)
Palatability issue formally captured and linked with outcome(s)	48 (21%)
Palatability issue formally captured but no link with outcome(s)	48 (21%)
Palatability issue formally captured with potential link to outcome(s)	19 (8%)
General experiences of patient, caregiver, and/or HCP only	5 (2%)

Table S7 Disease areas reported in the 225 studies by WHO ICD-11 Classification

ICD-11 Classification by Chapter	N (%)
**01 Certain infectious or parasitic diseases**	**46 (20.44%)**
Human immunodeficiency virus (HIV) infection	28
Malaria	7
Tuberculosis (TB)	4
Giardiasis	2
Chronic hepatitis C virus (HCV) infection	2
Cholera (oral vaccination)	1
Bacterial cellulitis, erysipelas or lymphangitis (bacterial skin infections)	1
Amoebiasis	1
**02 Neoplasms**	**3 (1.3%)**
Gliomas of brain and other solid tumours	2
Acute lymphoblastic leukaemia	1
**03 Diseases of the blood or blood-forming organs**	**14 (6.2%)**
Iron overload diseases (in transfusion dependent hematologic disorders)	8
Sickle cell disorders or other haemoglobinopathies	3
Iron deficiency anaemia	2
Beta thalassaemia	1
**04 Diseases of the immune system**	**2 (0.9%)**
Allergic or hypersensitivity conditions	2
**05 Endocrine, nutritional or metabolic diseases**	**18 (8.0%)**
Dehydration (in diarrhoea or burn injury)	6
Urea cycle disorders (inborn errors of metabolism)	5
Vitamin D deficiency	4
Homocystinuria (Inborn errors of metabolism)	1
Maple-syrup-urine disease (Inborn errors of metabolism)	1
Hypercholesterolemia (disorders of lipoprotein metabolism or certain specified lipidaemias)	1
**06 Mental, behavioural or neurodevelopmental disorders**	**2 (0.9%)**
Developmental learning disorder	1
Autism spectrum disorders	1
**08 Diseases of the nervous system**	**4 (1.8%)**
Epilepsy or seizures	4
**10 Diseases of the ear or mastoid process**	**9 (4.0%)**
Acute otitis media (bacterial ear infection)	9

ICD-11 Classification by Chapter	N (%)
**11 Diseases of the circulatory system**	**6 (2.7%)**
Hypertensive renal disease	3
Venous thromboembolism	2
Hypertension	1
**12 Diseases of the respiratory system**	**15 (6.7%)**
Asthma	6
Cystic fibrosis	2
Acute pharyngitis (upper respiratory tract disorders)	2
Pharyngotonsillitis (upper respiratory tract disorders)	1
Interstitial lung diseases associated with metabolic diseases	1
Pneumonia (lung infections)	1
Asthma / Croup	1
Asthma / Wheeze	1
**13 Diseases of the digestive system**	**20 (8.9%)**
Functional constipation of infants, toddlers or children	7
Dental caries	5
Oral mucositis	2
Gastro-oesophageal reflux disease	2
Eosinophilic oesophagitis	1
Liver diseases (Wilson disease, autoimmune liver disease, and transplant)	1
Dental caries / Gingival diseases	1
Inflammatory bowel diseases	1
**14 Diseases of the skin**	**1 (0.4%)**
Plaque psoriasis	1
**15 Diseases of the musculoskeletal system or connective tissue**	**3 (1.3%)**
Juvenile idiopathic arthritis	3
**16 Diseases of the genitourinary system**	**4 (1.8%)**
Renal tubular acidosis	2
Neuromuscular dysfunction of bladder	1
Neuromuscular dysfunction of bladder / Overactive bladder	1
**19 Certain conditions originating in the perinatal period**	**2 (0.9%)**
Neonatal hypomagnesaemia	2
**21 Symptoms, signs or clinical findings, not elsewhere classified**	**19 (8.4%)**
Pain	5
Pain / Fever	4

ICD-11 Classification by Chapter	N (%)
Nausea or vomiting (chemotherapy induced, post-operative or in acute gastroenteritis)	4
Fever	2
Diarrhoea	2
Wheeze	1
Cough	1
**22 Injury, poisoning or certain other consequences of external causes**	**12 (5.3%)**
Failure or rejection of transplanted organs or tissues: Bone marrow, heart, hematopoietic stem cell transplant, kidney, or liver	10
Poisoning (harmful effects of substances)	2
**X Extension codes: Medicaments**	**15 (6.7%)**
Medical procedure - Sedation and premedication	10
Diagnostics - Laxatives for colonoscopy	3
Diagnostics - Contrast Media	2
**Bacterial infections, Multiple indications**	**15 (6.7%)**
Includes studies using antimicrobials for various indications such as:	
01 Certain infectious or parasitic diseases	
Bacterial cellulitis, erysipelas or lymphangitis (bacterial skin infections)	
10 Diseases of the ear or mastoid process	
Acute otitis media (bacterial ear infection)	
12 Diseases of the respiratory system	
Acute bronchitis (lung infections)	
Acute laryngitis or tracheitis (upper respiratory tract disorders)	
Acute laryngopharyngitis (upper respiratory tract disorders)	
Acute nasopharyngitis (upper respiratory tract disorders)	
Acute pharyngitis (upper respiratory tract disorders)	
Acute sinusitis (upper respiratory tract disorders)	
Acute tonsillitis (upper respiratory tract disorders)	
Bronchitis (certain lower respiratory tract diseases)	
Infection prevention (in chemotherapy)	
Lung infections (in cystic fibrosis)	
Pharyngotonsillitis (upper respiratory tract disorders)	
Pneumonia (lung infections)	
13 Diseases of the digestive system	
Dental abscess (diseases of pulp or periapical tissues)	
16 Diseases of the genitourinary system	
Acute pyelonephritis	
Urinary tract infection, site not specified New	

ICD-11 Classification by Chapter	N (%)
21 Symptoms, signs or clinical findings, not elsewhere classified	
Pleurisy (symptoms or signs involving the respiratory system)	
**Non-specific disease area**	**15 (6.7%)**
Non-specific disease area	**15 (6.7%)**

Table S8 Drugs and medicinal products reported in the 225 studies by WHO ATC classification

**ATC Anatomical Main Group (1st Level)** Pharmacological Subgroup (3rd Level)	**ATC Code**	**Chemical Substance**	**N**	**References**
**A ALIMENTARY TRACT AND METABOLISM**				
A01A Stomatological Preparations	A01AB03	Chlorhexidine	4	Cheng 2004<br>Jain 2013<br>Nezam 2022<br>Splieth 2000
	A01AA01	Sodium fluoride	1	Berg 2006
	A01AD02	Benzydamine	1	Cheng 2004
	A01AD11	Enzyme containing mouthwash	1	Hoffstedt 2023
A02A Antacids	A02AH	Antacids with sodium bicarbonate	1	Claes 2014
A02B Drugs for peptic ulcer and gastro-oesophageal reflux disease (GORD)	A02BA02	Ranitidine	2	Ameen 2006<br>Venables 2015b
	A02BC01	Omeprazole	1	Venables 2015b
	A02BC04	Rabeprazole	1	Zannikos 2011
A03F Propulsives	A03FA03	Domperidone	1	Venables 2015b
A04A Antiemetics and antinauseants	A04AA01	Ondansetron	4	Cohen 2005<br>Ibrahim 2016<br>Nathan 2006<br>Stevens 1996
	A04AD05	Metopimazine	1	Nathan 2006
A06A Drugs for constipation	A06AD15	Macrogol	9	Imanieh 2022<br>Nimrouzi 2015<br>Rendeli 2006<br>Saneian 2012<br>Savino 2012<br>Sondheimer 1991<br>Venables 2015a<br>Venables 2015b<br>Voskuijl 2004
	A06AD11	Lactulose	5	Kokke 2008<br>Rendeli 2006<br>Saneian 2012<br>Venables 2015b<br>Voskuijl 2004
	A06AD65	Macrogol, combinations	3	Baker 2021

ATC Anatomical Main Group (1st Level)<br>Pharmacological Subgroup (3rd Level)	ATC Code	Chemical Substance	N	References
				Matsumoto 2022<br>Savino 2012
	A02AA04	Magnesium hydroxide	1	Saneian 2012
A07B Intestinal adsorbents	A07BA01	Medicinal charcoal	2	Dagnone 2002<br>Osterhoudt 2004
A07C Electrolytes with carbohydrates	A07CA	Oral rehydration salt formulations	7	Baker 2021<br>Caglayan 1989<br>Freedman 2010<br>Gyedu 2023<br>Nizami 1996<br>Pichichero 1990<br>Touchette 1994
A07E Intestinal anti-inflammatory agents	A07EA06	Budesonide	1	Warzecha 2022
A09A Digestives, including enzymes	A09AA02	Multienzymes (lipase, protease etc.)	2	Christiansen 2014<br>VandeVijver 2011
A11B Multivitamins, plain	A11BA	Multivitamins	1	Tiengkate 2022
A11C Vitamin A and D, including combinations of the two	A11CC	Vitamin D and analogues	5	Bartoli 2006<br>Claes 2014<br>Lava 2011<br>Penagini 2017<br>Pronzini 2008
A12B Potassium	A12BA30	Potassium (different salts in combination)	1	Bertholet-Thomas 2021
A12C Other mineral supplements	A12CB01	Zinc sulfate	3	Nasrin 2005<br>Nuzhat 2022<br>Suchismita 2023
	A12CC	Magnesium	2	Mettey 1990
	A12CB02	Zinc gluconate	1	Macknin 1998
A16A Other alimentary tract and metabolism products	A16AX03	Sodium phenylbutyrate	3	Kibleur 2014<br>Shchelochkov 2016<br>Yeowell 2021
	A16AX11	Sodium benzoate	3	Gaskell 2016

ATC Anatomical Main Group (1st Level)<br>Pharmacological Subgroup (3rd Level)	ATC Code	Chemical Substance	N	References
				Maines 2020<br>Yeowell 2021
	A16AA03	Glutamine	1	Gotesman 2020
	A16AA06	Betaine	1	Valayannopoulos 2019
	A16AA	Isoleucine (amino acid)	1	Goyanes 2019
	A16AA	L-arginine (amino acid)	1	Gaskell 2016
**B BLOOD AND BLOOD FORMING ORGANS**				
B01A Antithrombotic agents	B01AF01	Rivaroxaban	1	Young 2020
	B01AC24	Ticagrelor	1	DunivaInusa 2021
B03A Iron preparations	B03AA	Iron bivalent, oral preparations	3	DelRosso 2020<br>Powers 2020<br>Venables 2015b
B03B Vitamin B12 and folic acid	B03BB01	Folic acid	1	Venables 2015b
**C CARDIOVASCULAR SYSTEM**				
C08C Selective calcium channel blockers with mainly vascular effects	C08CA01	Amlodipine	2	Milani 2010<br>vanderVossen 2020
	C08CA13	Lercanidipine	1	Milani 2010
C09C Angiotensin II receptor blockers (ARBs), plain	C09CA01	Losartan	1	Meier 2007
	C09CA03	Valsartan	1	Meier 2007
	C09CA04	Irbesartan	1	Meier 2007
	C09CA06	Candesartan	1	Meier 2007
	C09CA07	Telmisartan	1	Meier 2007
C10A Lipid modifying agents, plain	C10AC01	Colestyramine	1	Liacouras 1993
**G GENITO URINARY SYSTEM AND SEX HORMONES**				
G04B Urologicals	G04BD12	Mirabegron	2	Baka-Ostrowska 2021<br>Rittig 2020

ATC Anatomical Main Group (1st Level)<br>Pharmacological Subgroup (3rd Level)	ATC Code	Chemical Substance	N	References
**H SYSTEMIC HORMONAL PREPARATIONS, EXCL. SEX HORMONES AND INSULINS**				
H02A Corticosteroids for systemic use, plain	H02AB06	Prednisolone	13	Aljebab 2018<br>Cronin 2016<br>Dawson 1993<br>Hames 2008<br>Haslund-Krog 2022<br>Mistry 2018<br>Parshuram 1998<br>Suchismita 2023<br>Taylor 2016<br>Tucker 2002<br>Venables 2015a<br>Venables 2015b<br>Venables 2018
	H02AB02	Dexamethasone	2	Aljebab 2018<br>Hames 2008
H03A Thyroid preparations	H03AA01	Levothyroxine	1	Venables 2015b
**J ANTIINFECTIVES FOR SYSTEMIC USE**				
J01C Beta-lactam antibacterials, penicillins	J01CR02	Amoxicillin and beta-lactamase inhibitor	12	Angelilli 2000<br>Block 2006<br>Cifaldi 2004<br>Cohen 2009<br>Holas 2005<br>Khurana 1996<br>Mistry 2018<br>Phipps 1990<br>Powers 1996<br>Powers 2000<br>Steele 2002<br>Toscani 2000
	J01CA04	Amoxicillin	5	Angwa 2020<br>Cohen 2009<br>Dagan 1994<br>Holas 2005<br>Mistry 2018
	J01CE02	Phenoxymethylpenicillin	5	Bagger-Sjoback 1989<br>Bergene 2019<br>Greenwood 2015<br>Uhari 1986<br>Venables 2015b

ATC Anatomical Main Group (1st Level)<br>Pharmacological Subgroup (3rd Level)	ATC Code	Chemical Substance	N	References
	J01CF05	Flucloxacillin	4	Elgammal 2022<br>Greenwood 2015<br>Rouse 2017<br>Venables 2015a
	J01CA02	Pivampicillin	1	Jahnsen 1987
	J01CF02	Cloxacillin	1	Matsui 1996
J01D Other beta-lactam antibacterials	J01DC10	Cefprozil	6	Angelilli 2000<br>Holas 2005<br>Powers 1996<br>Powers 2000<br>Steele 2002<br>Toscani 2000
	J01DD15	Cefdinir	6	Block 2005<br>Block 2006<br>Cifaldi 2004<br>Holas 2005<br>Powers 2000<br>Steele 2002
	J01DD08	Cefixime	4	Angelilli 2000<br>Powers 1996<br>Steele 2002<br>Toscani 2000
	J01DC02	Cefuroxime	3	Dagan 1994<br>Pichichero 1990<br>Steele 2002
	J01DC08	Loracarbef	3	ManfordGoochIII 1999<br>Powers 1996<br>Steele 2002
	J01DD13	Cefpodoxime	3	Cohen 2009<br>Powers 1996<br>Steele 2002
	J01DC04	Cefaclor	2	Dagan 1994<br>Steele 2002
	J01DB01	Cefalexin	1	Matsui 1996
J01E Sulfonamides and trimethoprim	J01EE01	Sulfamethoxazole and trimethoprim	4	Dagan 1994<br>Saito 1999<br>Venables 2015a<br>Venables 2015b
	J01EA01	Trimethoprim	1	Venables 2015b

ATC Anatomical Main Group (1st Level)<br>Pharmacological Subgroup (3rd Level)	ATC Code	Chemical Substance	N	References
J01F Macrolides, lincosamides and streptogramins	J01FA10	Azithromycin	8	Angelilli 2000<br>Block 2005<br>Holas 2005<br>Khurana 1996<br>Porteous 1997<br>Powers 2000<br>Steele 2002<br>Toscani 2000
	J01FA09	Clarithromycin	7	Abu-Khalaf 2018<br>Elgammal 2022<br>ManfordGoochIII 1999<br>Mistry 2018<br>Powers 1996<br>Steele 2002<br>Venables 2015a
	J01FA01	Erythromycin	2	Matsui 1996<br>Uhari 1986
	J01FA06	Roxithromycin	1	Moniot-Ville 1998
J01M Quinolone antibacterials	J01MA12	Levofloxacin	1	Purchase 2019
J01X Other antibacterials	J01XC01	Fusidic acid	1	Matsui 1996
	J01XE01	Nitrofurantoin	1	Venables 2015a
	J01XB02	Polymyxin B	1	Saito 1999
	J01XD02	Tinidazole	1	Escobedo 2008
J04A Drugs for treatment of tuberculosis	J04AB02	Rifampicin	1	Venables 2015a
	J04AK06	Delamanid	1	Garcia-Prats 2022
	J04AM05	Rifampicin, pyrazinamide and isoniazid	1	Wademan 2019
	J05AR10	Lopinavir and ritonavir	10	Coetzee 2015<br>Coetzee 2016<br>Giralt 2019<br>Kekitiinwa 2016<br>Lin 2011<br>Murnane 2017<br>Pasipanodya 2018<br>Purchase 2016<br>Thomson 2015

ATC Anatomical Main Group (1st Level)<br>Pharmacological Subgroup (3rd Level)	ATC Code	Chemical Substance	N	References
				Venables 2015a
	J05AE03	Ritonavir	9	Allen 2003<br>Chadwick 2005<br>Davies 2008<br>Frange 2018<br>Goode 2003<br>Lin 2011<br>Melvin 1997<br>Reddington 2000<br>VanDyke 2002
	J05AF01	Zidovudine	5	PENTA 1999<br>Bunupuradah 2006<br>Goode 2003<br>Lin 2011<br>Nahirya-Ntege 2012
	J05AE04	Nelfinavir	4	Goode 2003<br>Lin 2011<br>Reddington 2000<br>VanDyke 2002
	J05AF05	Lamivudine	4	Bunupuradah 2006<br>Coetzee 2016<br>Lin 2011<br>Nahirya-Ntege 2012
	J05AF06	Abacavir	3	Coetzee 2016<br>Lin 2011<br>Nahirya-Ntege 2012
	J05AG01	Nevirapine	3	Bunupuradah 2006<br>Lin 2011<br>Nahirya-Ntege 2012
	J05AE02	Indinavir	2	Bunupuradah 2006<br>Lin 2011
	J05AF02	Didanosine	2	Bunupuradah 2006<br>Lin 2011
	J05AF04	Stavudine	2	Bunupuradah 2006

ATC Anatomical Main Group (1st Level)<br>Pharmacological Subgroup (3rd Level)	ATC Code	Chemical Substance	N	References
				Lin 2011
	J05AG03	Efavirenz	2	Bunupuradah 2006<br>Lin 2011
	J05AE01	Saquinavir	1	Lin 2011
	J05AE05	Amprenavir	1	Lin 2011
	J05AF03	Zalcitabine	1	Lin 2011
	J05AJ01	Raltegravir	1	Nachman 2014
	J05AP08	Sofosbuvir	1	Rosenthal 2020
	J05AP51	Sofosbuvir and ledipasvir	1	Schwarz 2020
	J05AR	Abacavir/ Lamivudine/Lopinavir/Ritonavir	1	Rotsaert 2023
	J05AR01	Zidovudine and lamivudine	1	Lin 2011
	J05AR14	Darunavir and cobicistat	1	Dalton 2019
J07A Bacterial vaccines	J07AE02	Cholera, live attenuated	1	Lagos 1999
**L ANTINEOPLASTIC AND IMMUNOMODULATING AGENTS**				
L01B Antimetabolites	L01BB02	Mercaptopurine	2	Mulla 2016<br>Venables 2015b
L01E Protein kinase inhibitors	L01EE01	Trametinib	1	Escoda 2017
L01X Other antineoplastic agents	L01XD04	Aminolevulinic acid	1	Wataya 2017
	L01XX05	Hydroxycarbamide (or Hydroxyurea)	1	Prajapati 2022
L04A Immunosuppressants	L04AD01	Ciclosporin	2	Claes 2014<br>Tucker 2002
	L04AD02	Tacrolimus	2	Claes 2014<br>Gamston 2013
	L04AA32	Apremilast	1	Paller 2019
	L04AF01	Tofacitinib	1	Ruperto 2017
	L04AX01	Azathioprine	1	Tucker 2002
	L04AX03	Methotrexate	1	Scheuern 2017
**M MUSCULO-SKELETAL SYSTEM**				
M01A Anti-inflammatory and antirheumatic products, non-steroids	M01AE01	Ibuprofen	9	Al-Ani 2016<br>Barnett 2020<br>Celebi 2009<br>Klingmann 2022

ATC Anatomical Main Group (1st Level)<br>Pharmacological Subgroup (3rd Level)	ATC Code	Chemical Substance	N	References
				McIntyre 1996<br>Mistry 2018<br>Smith 2013<br>Steans 1990<br>Venables 2015b
	M01AE03	Ketoprofen	2	Celebi 2009<br>Kokki 2000
**N NERVOUS SYSTEM**				
N02A Opioids	N02AA01	Morphine	2	Jamieson 2021<br>Venables 2015a
	N02AX02	Tramadol	1	Yoo 2022
	N02AX06	Tapentadol	1	Beuter 2019
	N07BC06	Diamorphine	1	Jamieson 2021
N02B Other analgesics and antipyretics	N02BE01	Paracetamol	8	Celebi 2009<br>Klingmann 2022<br>Kolmen 1995<br>McIntyre 1996<br>Mistry 2018<br>Smith 2013<br>Venables 2015a<br>Venables 2015b
	N02BE51	Paracetamol, combinations excl. psycholeptics	1	Jaffe 1983
N03A Antiepileptics	N03AG01	Valproic acid	3	Cloyd 1992<br>Kiryluk 2013<br>Venables 2015b
	N03AX14	Levetiracetam	2	Joanne 2018<br>Venables 2015b
	N03AF01	Carbamazepine	1	Venables 2015b
N05C Hypnotics and sedatives	N05CD08	Midazolam	8	Almenrader 2007<br>Cote 2002<br>Isik 2008<br>Khalil 2003<br>Kumar 2012<br>Marshall 2000<br>Riva 1997<br>Xin 2022
	N05CC01	Chloral hydrate	4	Chung 2000<br>Tiengkate 2022

ATC Anatomical Main Group (1st Level)<br>Pharmacological Subgroup (3rd Level)	ATC Code	Chemical Substance	N	References
				Venables 2015a<br>Zhang 2016
	N05CA01	Pentobarbital	1	Chung 2000
N07B Drugs used in addictive disorders	N07BB04	Naltrexone	1	Kolmen 1995
**P ANTIPARASITIC PRODUCTS, INSECTICIDES AND REPELLENTS**				
P01A Agents against amoebiasis and other protozoal diseases	P01AB01	Metronidazole	1	Canete 2010
	P01AB07	Secnidazole	1	Padilla 2000
P01B Antimalarials	P01BA01	Chloroquine	4	Ansah 2001<br>Braga 2015<br>Canete 2010<br>Kolaczinski 2006
	P01BF01	Artemether and lumefantrine	3	Abdulla 2010<br>Ewing 2015<br>Kedenge 2013
	P01BA03	Primaquine	1	Braga 2015
	P01BC01	Quinine	1	Hofmanova 2020
	P01BD51	Pyrimethamine, combinations	1	Kolaczinski 2006
	P01BF07	Artemisinin and piperaquine	1	Ewing 2015
**R RESPIRATORY SYSTEM**				
R01B Nasal decongestants for systemic use	R01BA52	Pseudoephedrine, combinations	1	Jaffe 1983
R02A Throat preparations	R02AA03	Dichlorobenzyl alcohol	1	Thompson 2013
R03D Other systemic drugs for obstructive airway diseases	R03DA04	Theophylline	1	Rasul 2001
R05D Cough suppressants, excluding combinations with expectorants	R05DA04	Plain codeine (classified in this group even when used as an analgesic)	1	Smith 2013
R06A Antihistamines for systemic use	R06AX12	Terfenadine	1	Holmberg 1994
	R06AX13	Loratadine	1	Holmberg 1994
**V VARIOUS**				
V01A Allergens	V01AA20	Allergen extracts (various)	1	Pajno 2012

ATC Anatomical Main Group (1st Level)<br>Pharmacological Subgroup (3rd Level)	ATC Code	Chemical Substance	N	References
V03A All other therapeutic products	V03AC03	Deferasirox	6	Goldberg 2013<br>Holloway 2018<br>Horodniceanu 2017<br>Taher 2018<br>Tsouana 2015<br>Viprakasit 2023
	V03AE01	Polystyrene sulfonate	1	Tiengkate 2022
	V03AB26	Methionine	1	Querin 2023
V08A X-ray contrast media, iodinated	V08AA01	Diatrizoic acid	1	Venables 2015a
	V08AA05	Ioxitalamic acid	1	Tannous 1991
V08B X-ray contrast media, non-iodinated	V08BA01	Barium sulfate with suspending agents	1	Dillman 2018
**OTHER INTERVENTIONAL PRODUCTS**				
Beverage		Breeza as negative contrast media	1	Dillman 2018
		Fiber Mixture	1	Kokke 2008
Herbal medicine		Descurainia sophia seeds	1	Nimrouzi 2015
		Liquorice root extract	1	Jain 2013
		Qingre Huazhi Decoction	1	Hou 2022
		Rosa Damascena	1	Imanieh 2022
		Terminalia chebula	1	Palit 2016
Medical device		Muco-adhesive oral rinse	1	Gibson 2010
Medicinal substance, not classified		Isobutyramide	1	Reich 2000
		Quinfamide	1	Padilla 2000
Probiotic		Streptococcus salivarius K12 strain	1	DiPierro 2014
